# Spatially resolved multimarker evaluation of CD274 (PD-L1)/PDCD1 (PD-1) immune checkpoint expression and macrophage polarisation in colorectal cancer

**DOI:** 10.1038/s41416-023-02238-6

**Published:** 2023-03-31

**Authors:** Hanna Elomaa, Maarit Ahtiainen, Sara A. Väyrynen, Shuji Ogino, Jonathan A. Nowak, Mai Chan Lau, Olli Helminen, Erkki-Ville Wirta, Toni T. Seppälä, Jan Böhm, Jukka-Pekka Mecklin, Teijo Kuopio, Juha P. Väyrynen

**Affiliations:** 1 Department of Biological and Environmental Science, University of Jyväskylä, Jyväskylä, Finland; 2 Department of Education and Research, Hospital Nova of Central Finland, Well Being Services County of Central Finland, Jyväskylä, Finland; 3 Department of Pathology, Hospital Nova of Central Finland, Well Being Services County of Central Finland, Jyväskylä, Finland; 4 Department of Internal Medicine, Oulu University Hospital, Oulu, Finland; 5 Program in Molecular Pathological Epidemiology, Department of Pathology, Brigham and Women’s Hospital and Harvard Medical School, Boston, MA USA; 6 Department of Epidemiology, Harvard T.H. Chan School of Public Health, Boston, MA USA; 7 Broad Institute of MIT and Harvard, Cambridge, MA USA; 8 Cancer Immunology and Cancer Epidemiology Programs, Dana-Farber Harvard Cancer Center, Boston, MA USA; 9 Institute of Molecular Cell Biology, Agency of Science, Technology and Research (A*STAR), Singapore, Singapore; 10 Translational Medicine Research Unit, Medical Research Center Oulu, Oulu University Hospital, and University of Oulu, Oulu, Finland; 11 Department of Gastroenterology and Alimentary Tract Surgery, Tampere University Hospital, Tampere, Finland; 12 Faculty of Medicine and Health Technology, Tampere University and Tays Cancer Center, Tampere University Hospital, Tampere, Finland; 13 Department of Gastrointestinal Surgery, Helsinki University Central Hospital, University of Helsinki, Helsinki, Finland; 14 Applied Tumor Genomics, Research Program Unit, University of Helsinki, Helsinki, Finland; 15 Faculty of Sport and Health Sciences, University of Jyväskylä, Jyväskylä, Finland

**Keywords:** Cancer microenvironment, Prognostic markers, Tumour immunology, Colorectal cancer

## Abstract

**Background:**

The CD274 (PD-L1)/PDCD1 (PD-1) immune checkpoint interaction may promote cancer progression, but the expression patterns and prognostic significance of PD-L1 and PD-1 in the colorectal cancer microenvironment are inadequately characterised.

**Methods:**

We used a custom 9-plex immunohistochemistry assay to quantify the expression patterns of PD-L1 and PD-1 in macrophages, T cells, and tumour cells in 910 colorectal cancer patients. We evaluated cancer-specific mortality according to immune cell subset densities using multivariable Cox regression models.

**Results:**

Compared to PD-L1^–^ macrophages, PD-L1^+^ macrophages were more likely M1-polarised than M2-polarised and located closer to tumour cells. PD-L1^+^ macrophage density in the invasive margin associated with longer cancer-specific survival [*P*_trend_ = 0.0004, HR for the highest vs. lowest quartile, 0.52; 95% CI: 0.34–0.78]. T cell densities associated with longer cancer-specific survival regardless of PD-1 expression (*P*_trend_ < 0.005 for both PD-1^+^ and PD-1^–^ subsets). Higher densities of PD-1^+^ T cell/PD-L1^+^ macrophage clusters associated with longer cancer-specific survival (*P*_trend_ < 0.005).

**Conclusions:**

PD-L1^+^ macrophages show distinct polarisation profiles (more M1-like), spatial features (greater co-localisation with tumour cells and PD-1^+^ T cells), and associations with favourable clinical outcome. Our comprehensive multimarker assessment could enhance the understanding of immune checkpoints in the tumour microenvironment and promote the development of improved immunotherapies.

## Background

Colorectal cancer is the second leading cause of cancer death worldwide with over 900,000 deaths in 2020 [1]. The assessment of cancer prognosis and treatment is mainly based on the tumour extent and tumour morphology, but the rapidly increasing knowledge on the significance of tumour immune contexture has led to the development of improved immune-related prognostic markers and effective anticancer immunotherapies [2]. In addition to the quantity of immune cells, the activity of immunoregulatory signalling pathways, such as the co-inhibitory pathway of programmed death ligand 1 (PD-L1, CD274) and its receptor programmed death 1 (PD-1, PDCD1), may affect cancer progression. Hereinafter, CD274 and PDCD1 are referred as PD-L1 and PD-1 due to the ubiquity of these “colloquial” protein names. PD-L1 is mainly expressed in macrophages [3], whereas PD-1 is mainly expressed in T cells [4]. The expression of PD-L1 and PD-1 is often upregulated in cancer and their interaction may lead to immunosuppression through T cell exhaustion, thus promoting tumour growth [4]. However, the prognostic significance of immune checkpoint protein expression in many tumour types, including colorectal cancer, has remained controversial [5].

Macrophages are inflammatory cells which have shown to associate with cancer progression [6]. They are commonly categorised into classically activated, M1-polarised, and alternatively activated, M2-polarised macrophages, which differ by their surface antigens, cytokine secretion profiles, and physiological functions. However, instead of two completely distinct subtypes, macrophages are considered to form a continuum of phenotypes that may gradually change their polarisation towards M1-like or M2-like and simultaneously express phenotypic markers for both subtypes. In the tumour microenvironment, M1-like macrophages are thought to have pro-inflammatory effect and be more prevalent in early-stage cancer, whereas the proportion of anti-inflammatory M2-like macrophages increases along cancer progression [6]. Higher M1/M2-like macrophage ratio has been thought to associate with improved cancer-specific survival [7]. The expression patterns of immune checkpoints in macrophages and their clinical value are poorly established in colorectal cancer.

In this study, we used multiplex immunohistochemistry and machine learning-based image analysis to comprehensively characterise PD-L1 and PD-1 immune checkpoint expression in M1-like and M2-like macrophages, T cells, and tumour cells in a large, population-based colorectal cancer cohort of 910 patients. Our primary aim was to (i) evaluate the expression patterns and prognostic significance of PD-L1 and PD-1 in immune cells and tumour cells. As secondary aims, we (ii) clarified the associations between immune checkpoint expression and tumour characteristics, and (iii) investigated the infiltration patterns and prognostic role of M1-like and M2-like polarised macrophages. We hypothesised low expression of PD-L1 and PD-1 immune checkpoints and higher density of M1-like macrophages to associate with favourable colorectal cancer outcome.

## Methods

### Study population

The study was based on a cohort of 1343 colorectal cancer patients, who underwent a resection for primary colon or rectum carcinoma between January 1, 2000 and December 31, 2015 in Central Finland Central Hospital. The population of the area was on average 270,000 during the study period [8]. The clinical, histopathological, and follow-up data were retrospectively collected from the pathology registry and clinical records of Central Finland Central Hospital. All tumours were screened for DNA mismatch repair (MMR) deficiency and *BRAF* V600E mutation status with immunohistochemistry [9]. Histological tumour parameters, including tumour differentiation and lymphovascular invasion, were re-evaluated from hematoxylin and eosin-stained whole slides by the study pathologist (J.P.V.). All histological data were analysed blinded to clinical data. We excluded patients who died within 30 days after surgery (*N* = 40) or received any preoperative oncological treatments (radiotherapy, chemotherapy, or chemoradiotherapy) (*N* = 243) due to their potential influences on tumour characteristics or immune response [10]. After applying further exclusion criteria of unsuccessful multiplex immunohistochemistry staining or inadequate tumour tissue of both the tumour centre and the invasive margin in tissue microarrays (*N* = 150), the final cohort comprised samples of 910 colorectal cancer patients.

### Multiplex immunohistochemistry

Tissue microarrays were constructed by selecting four 1-mm diameter cores from each tumour [9]. We designed a 9-plex immunohistochemistry panel to identify macrophages with CD68 and CD163, T cells with CD3, and tumour cells with KRT (keratin). PD-L1 and PD-1 immune checkpoint molecules and four macrophage polarisation markers (CD86, CD163, HLA-DR, MRC1) were included in the assay.

The multiplex immunohistochemistry staining was done with Bond-III automated IHC stainer (Leica Biosystems, Buffalo Grove, IL, USA) and Bond Refine Detection kit (DS9800, Leica Biosystems). We used sections of 3.5 µm. Candidate antibodies and suitable dilutions were optimised using conventional immunohistochemistry with 3,3′-Diaminobenzidine (DAB) chromogen in a test tissue microarray consisting of normal colorectal mucosa, colorectal cancer tissue, and tonsil tissue. These antibodies were then combined into a multiplex immunohistochemistry assay. The correspondence of staining patterns of multiplex and conventional immunohistochemistry were visually confirmed in serial sections of a test microarray, and the correspondence was further quantified by manually annotating cells in 10 respective regions (size 200 × 200 µm) of multiplex and conventional immunohistochemistry (Supplementary Fig. S1). The multiplex staining was conducted using a previously validated, cyclic method that uses 3-Amino-9-Ethylcarbazole (AEC) as the chromogen [11]. The workflow for the staining is shown in Supplementary Fig. S2 and the selected monoclonal antibodies along with their dilutions and antigen retrieval conditions are listed in Supplementary Table S1. We used AEC^+^ high sensitivity substrate (K3469, Dako, Glostrup, Denmark) as the chromogen. After each cycle, the slides were mounted with VectaMount AQ Aqueous Mounting Medium (H-5501, Vector Laboratories, Newark, CA, USA), scanned with 20× objective of NanoZoomer XR (Hamamatsu Photonics, Hamamatsu City, Japan, resolution 0.45 µm/pixel), de-stained with ethanol, and heated to remove the primary and secondary antibodies.

### Image analysis

**Fig. 1:**
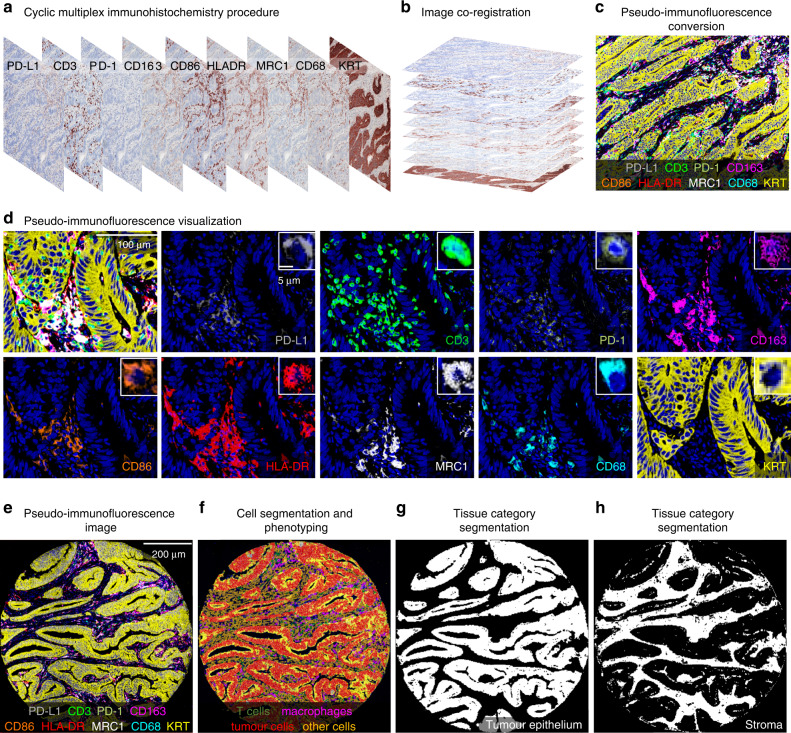
Multiplex immunohistochemistry panel and image analysis. **a** Digitised multiplex immunohistochemistry image from each staining cycle for one tumour. **b**, **c** Image co-registration based on aligning the hematoxylin layers to merge individual images into one 10-channel pseudo-immunofluorescence image. **d** example images showing the expression patterns of each marker merged and separately. **e**−**h** pseudo-immunofluorescence image (**e**), machine learning-based cell segmentation and phenotyping into T cells, macrophages, tumour cells, and other cells (**f**), and mask images of the categorisation of tissue into tumour epithelium and stroma (**g**, **h**).

The digitised images of multiplex immunohistochemistry slides were processed with QuPath (version 0.2.3) [12]. Tissue microarray cores were recognised with *TMA dearrayer* function and separated into single core images. We excluded cores which were folded, included minimal amount of tumour, were necrotic, or comprised less than 50% of the 1-mm diameter core area after all staining cycles. The single core images of all 9 staining cycles were stacked into one 10-channel pseudo-immunofluorescence image (with hematoxylin as the 10th channel) by aligning cell nuclei using the MultiStackReg macro (downloaded from http://bradbusse.net/downloads.html) in ImageJ/Fiji open-source software [13]. This macro enabled the co-registration of the images despite potential minor shifts in tissue during the staining cycles. The conversion of single images into a 10-plex pseudo-immunofluorescence image is illustrated in Fig. 1a–d. The staining intensities were consistent across tissue microarrays (Supplementary Fig. S3), indicating that the assay had performed uniformly.

The pseudo-immunofluorescence images were analysed with QuPath, utilising previously validated [14], supervised machine learning algorithms. We identified cells with *cell detection* function and calculated additional smoothed object features to improve subsequent cell phenotyping. Cells were phenotyped into T cells, macrophages, tumour cells, and other cells using the *object classifier* function built in QuPath, based on the random forests algorithm. For training, example cells were annotated as follows: (1) all CD3 expressing cells were phenotyped as T cells; (2) cells expressing CD68 [15] and/or CD163 [7] were identified as macrophages in line with a prior study [16]; (3) KRT expressing cells were phenotyped as tumour cells; and (4) cells negative for CD3, CD68, CD163 and KRT were classified as other cells. Cell data yielded phenotypes, marker intensities, and coordinates for each cell. To classify tissue categories, QuPath was trained to identify tissue segments of tumour epithelium and stroma using the built-in *pixel classifier* function. Necrotic regions, empty white space without any tissue, and regions of partial core loss (the hematoxylin staining from the first staining cycle not corresponding with the hematoxylin staining form the last staining cycle) were excluded. The workflow for cell and tissue analyses is described in Supplementary Fig. S4 and the example images from cell segmentation and tissue categorisation in QuPath are represented in Fig. 1e–h.

### Immune cell phenotyping and classification

Cell level data were further processed with RStudio (version 1.3.1093) and R statistical programming (version 4.0.3, R Core Team).

Macrophages were classified according to PD-L1 expression and M1/M2 polarisation state. To categorise macrophages according to their polarisation, we calculated a polarisation index for each macrophage consistent with a prior study [16]. First, we converted the intensities of all four macrophage markers into percentiles across all macrophages and calculated a polarisation index by reducing the intensities of M2-like macrophage markers from the intensities of M1-like macrophage markers [formula: (CD86 + HLA-DR) − (CD163 + MRC1); with marker names denoting intensity percentiles]. Using this formula, macrophages with higher polarisation index values were considered more M1-polarised, while those with lower values were considered more M2-polarised. For downstream analyses, the index values were divided into ordinal quartile categories (Q1–Q4) across all macrophages in 910 colorectal cancer cases. As in a prior study [16], macrophages in the lowest group (Q1) were classified as M1-like macrophages and those in the highest group (Q4) were classified as M2-like macrophages. Macrophages in the middle groups (Q2–Q3), covering 50% of the cells, were left unclassified to limit this analysis to the most strongly polarised M1-like and M2-like macrophages. For downstream survival analyses, we calculated the densities of various macrophage subsets in the tumour centre and the invasive margin and categorised the densities into ordinal quartile categories (Q1–Q4). For variables including over 25% zero densities, all zero values were categorised into the lowest group Q1. The remaining values were divided equally into groups Q2–Q4. Ordinal quartile categories were also similarly defined for other immune cell variables.

In addition to macrophages, PD-L1 expression was evaluated in tumour epithelial cells, based on the weighted histoscore method. For each cell, we classified the staining intensity (negative, weak, intermediate, or high), and for each case, we calculated the percentage (0–100%) of tumour cells within each category separately in the tumour centre and the invasive margin. In cases with multiple tissue microarray cores from one region, we calculated the mean values. Finally, PD-L1 histoscore was calculated as follows: PD-L1 histoscore = [(1 × percentage of weakly stained cells)+(2 × percentage of moderately stained cells)+(3 × percentage of strongly stained cells)]. The possible range for values was from 0 (all cells negative) to 300 (all cells strongly positive) [17]. For survival analyses, we calculated the mean histoscore for each tumour and categorised the tumours into negative or positive using a cut-off value of ≥5 for the positivity.

T cells were further subdivided into PD-1 positive or negative based on a fixed cut-off value for their cytoplasmic PD-1 staining intensity (40 intensity units).

To analyse spatial interactions between immune and tumour cells, we used the *spatstat* (2.2–0) package to calculate nearest neighbour distances (NNDs), which measure the distance from a specific point (e.g., macrophage) to its closest neighbour point of specific category (e.g., tumour cell). For visualisation, scaled intensities of macrophage polarisation markers as a function of NND from tumour cells were plotted with *ggplot2* (3.3.3) package using generalised additive model smoothing [formula y ~ s(x)]. For further spatial analyses, we calculated the density of PD-1^+^/PD-L1^+^ clusters defined as PD-1^+^ T cell located within 20-μm distance from the closest PD-L1^+^ macrophage. The radius was selected in order to identify cells with capability for direct cell-cell interaction consistent with prior reports [9, 18].

### Statistical analysis

Statistical analyses were performed in RStudio using packages *corrplot* (0.90), *forestplot* (2.0.1), *ggpubr* (0.4.0), *gmodels* (2.18.1), *spatstat* (2.1–0), *survival* (3.2–7), *survminer* (0.4.9), and *tidyverse* (1.3.1).

We used the Wilcoxon rank-sum test for dichotomous variables and the Kruskal–Wallis test for variables with three or more categories to evaluate the associations of continuous immune cell density variables with patient characteristics. The associations of categorical immune cell density variables with patient characteristics were tested with crosstabulation and the Chi-square test to evaluate the statistical significance. We examined the correlations between immune cell densities by calculating Spearman’s correlation coefficients.

As our main analysis, we used univariable and multivariable Cox proportion hazard regression models to measure hazard ratio (HR) point estimates and 95% confidence intervals (CIs) for cancer-specific and overall survival. Cancer-specific survival was considered as the primary survival endpoint, and it was defined as the time from surgery to colorectal cancer death or the end of follow-up. Overall survival was defined as the duration from surgery to death of any cause or the end of follow-up. The total number of deaths was 525 (58%) including 250 (31%) cancer-specific deaths. The median follow-up time for censored cases was 10.1 years (IQR 6.6–13.1). We limited the follow-up to 10 years, considering that most colorectal cancer deaths occur within that period. Schoenfeld residual plots supported the proportionality of hazards during most of the follow-up period up to 10 years. Multivariable models included the following pre-determined indicator variables (with the reference category listed first): sex (male, female), age (<65, 65–75, >75), year of operation (2000–2005, 2006–2010, 2011–2015), tumour location (proximal colon, distal colon, rectum), American Joint Committee on Cancer (AJCC) stage (I–II, III, IV), tumour grade (well/moderately differentiated, poorly differentiated), lymphovascular invasion (negative, positive), MMR status (proficient, deficient), *BRAF* status (wild-type, mutant). Kaplan–Meier method was used to visualise the estimates of cancer-specific survival, and the statistical significance was tested with the Log-rank test. A *P* value less than 0.005 was considered statistically significant, in accordance with the recommendation of an expert panel [19].

## Results

### Image analysis

We analysed 3190 tissue microarray cores from 910 colorectal cancer patients (mean 3.5 per patient, SD 0.69; tumour centre: mean 1.8, SD 0.39; invasive margin: mean 1.7, SD 0.51). The supervised machine learning algorithms yielded data for 21,503,560 cells including 9,136,506 tumour cells, 1,788,538 macrophages, and 1,582,095 T cells. Macrophages were further phenotyped into PD-L1^+^ and PD-L1^−^ and M1- and M2-like subpopulations. T cells were phenotyped into PD-1^+^ and PD-1^−^ subpopulations. The median immune cell densities in the tumour centre and the invasive margin were 512 and 808 cells/mm^2^ for all macrophages, 33 and 64 cells/mm^2^ for PD-L1^+^ macrophages and 390 and 591 cells/mm^2^ for T cells, respectively. Core-to-core correlations for immune cell densities were good or moderate in both the tumour centre and the invasive margin (Supplementary Fig. S5).

### PD-L1 expression patterns

PD-L1 expression was detected mainly in macrophages. Of macrophages, 20% were positive and 80% were negative for PD-L1. In macrophage subsets defined by four polarisation markers, PD-L1 expression was enriched in M1-like macrophages. Of PD-L1^+^ macrophages, 33% were M1-like, 16% were M2-like, and the remaining 51% were less strongly polarised mixed phenotype macrophages. Correspondingly, PD-L1^+^ macrophage density and M1-like macrophage density showed a moderate positive correlation (R = 0.54), while the correlation between the densities of PD-L1^+^ macrophages and M2-like macrophages was weak (R = 0.24) (Supplementary Fig. S6). Conversely, PD-L1^−^ macrophage densities showed a stronger correlation with M2-like macrophage densities (R = 0.67) than M1-like macrophage densities (R = 0.29). Median densities for both PD-L1^+^ and PD-L1^−^ macrophages were higher in tumour stromal (110/mm^2^ for PD-L1^+^ and 991/mm^2^ for PD-L1^−^) than intraepithelial regions (11.5/mm^2^ for PD-L1^+^ and 119/mm^2^ for PD-L1^−^).

The clinicopathological characteristics according to PD-L1^+^ and PD-L1^−^ macrophage densities are shown in Table 1. Higher density of PD-L1^+^ macrophages associated with proximal tumour location (*P* = 0.0012), low stage, poor tumour differentiation, absent lymphovascular invasion, MMR deficiency, and *BRAF* mutation (all *P* < 0.0001). Higher density of PD-L1^−^ macrophages associated with high stage (*P* = 0.0049). The associations of macrophage polarisation with clinicopathological characteristics are visualised in Supplementary Fig. S7.

**Table 1:** Demographic and clinical characteristics of colorectal cancer cases according to PD-L1^+^ and PD-L1^–^ macrophage densities.

Characteristic	Total *N*	Overall cell density (cells/mm^2^) Median (25–75th percentiles)			
		PD-L1^+^ macrophages	*P*	PD-L1^−^ macrophages	*P*
All cases	910 (100%)	59 (16–170)		560 (380–780)	
Sex			0.50		0.47
Male	464 (51%)	60 (17–160)		570 (390–790)	
Female	446 (49%)	59 (15–190)		560 (370–770)	
Age (years)			0.18		0.95
<65	247 (27%)	54 (15–160)		580 (360–790)	
65–75	331 (36%)	58 (15–150)		560 (390–750)	
>75	332 (36%)	74 (18–230)		550 (370–800)	
Tumour location			0.0012		0.19
Proximal colon	445 (49%)	74 (20–220)		580 (420–790)	
Distal colon	332 (36%)	51 (12–140)		530 (360–750)	
Rectum	133 (15%)	38 (15–160)		560 (360–750)	
AJCC stage			<0.0001		0.0049
I	151 (17%)	84 (19–210)		500 (340–670)	
II	342 (38%)	84 (26–210)		540 (390–790)	
III	301 (33%)	47 (11–130)		590 (420–800)	
IV	116 (13%)	26 (6.6–100)		650 (370–890)	
Tumour grade			<0.0001		0.73
Low-grade (well to moderately differentiated)	760 (84%)	55 (15–160)		560 (370–780)	
High-grade (poorly differentiated)	150 (16%)	104 (24–310)		570 (410–760)	
Lymphovascular invasion			<0.0001		0.20
No	719 (79%)	70 (21–190)		550 (380–770)	
Yes	191 (21%)	32 (6.5–100)		600 (380–820)	
MMR status			<0.0001		0.20
MMR proficient	772 (85%)	48 (12–150)		550 (370–780)	
MMR deficient	137 (15%)	190 (60–350)		590 (460–770)	
*BRAF* status			<0.0001		0.046
Wild-type	763 (84%)	52 (14–160)		550 (370–770)	
Mutant	147 (16%)	98 (37–290)		590 (440–790)	

*AJCC* American Joint Committee on Cancer, *MMR* mismatch repair.

In addition to macrophages, we investigated the PD-L1 expression in tumour cells using the histoscore method. Most of the tumours were negative for PD-L1 or showed very weak expression (histoscore < 5), and only 66 (7%) of the tumours were classified as PD-L1 positive (histoscore ≥ 5). Similar to high PD-L1^+^ macrophage density, PD-L1 positivity in tumour cells associated with proximal tumour location, poor differentiation, MMR deficiency and *BRAF* mutation (all *P* < 0.0001), but there were no associations with stage or lymphovascular invasion (Supplementary Table S2).

### PD-1 expression patterns

We evaluated PD-1 expression in T cells. Of all detected T cells, 22% were positive for PD-1. Higher T cell density associated with low stage [*P* = 0.00025 (tumour centre), *P* < 0.0001 (invasive margin)], high tumour grade (*P* < 0.0001, *P* = 0.0032), MMR deficiency (both *P* < 0.0001) and *BRAF* mutation (*P* < 0.0001, *P* = 0.062). The findings were mainly similar for PD-1^+^ and PD-1^−^ T cells (Supplementary Fig. S8). The density of PD-L1^+^ macrophages positively correlated with the densities of both PD-1^+^ T cells (R = 0.64) and PD-1^−^ T cells (R = 0.50) (Supplementary Fig. S6).

### Survival analyses

**Fig. 2:**
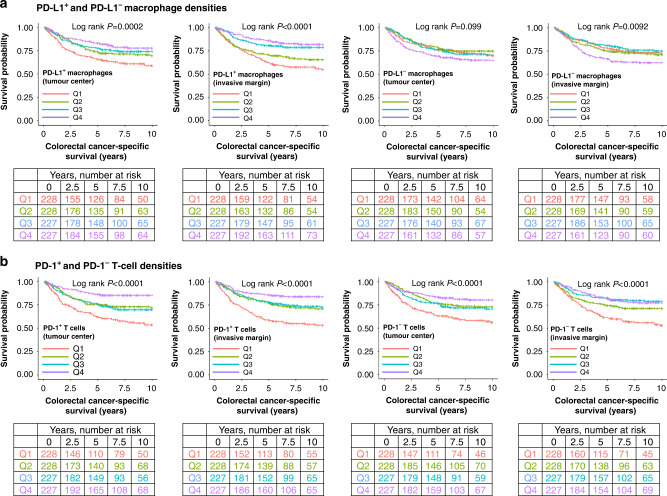
Kaplan–Meier survival analysis. Cancer-specific survival curves for the PD-L1^+^ and PD-L1^−^ macrophage (**a**) and PD-1^+^ and PD-1^−^ T cell (**b**) densities in the tumour centre and the invasive margin. The densities were divided into ordinal quartiles from low (Q1) to high (Q4). Statistical significance was determined with Log-rank test.

We first examined the prognostic role of total (CD68^+^/CD163^+^) macrophage population, which did not reach statistical significance either in the tumour centre or the invasive margin in univariable (Supplementary Fig. S9, Table 2) or multivariable analyses (Table 2) (all *P* > 0.005). When macrophages were classified according to their PD-L1 expression, higher density of PD-L1^+^ macrophages associated with improved cancer-specific survival in both the tumour centre and the invasive margin in univariable analyses (Fig. 2a, Table 2). The prognostic value remained significant in the invasive margin in multivariable analysis (*P*_trend_ = 0.0004, HR for Q4 vs. Q1 0.52, 95% CI 0.34–0.78) (Table 2). The full multivariable Cox regression models for PD-L1^+^ and PD-L1^−^ macrophage densities are shown in Supplementary Table S3. When tumour epithelial and stromal compartments were examined separately, higher PD-L1^+^ macrophage density associated with longer cancer-specific survival in both tumour intraepithelial and stromal compartments of the tumour centre and the invasive margin in univariable analyses (all *P*_trend_ < 0.005) (Supplementary Fig. S10, Supplementary Table S4). These associations remained significant in the invasive margin in multivariable analyses (Supplementary Table S4). Higher density of PD-L1^−^ macrophages in the invasive margin tended to associate with poor survival but did not reach statistical significance in either univariable or multivariable analysis (*P*_trend_ > 0.005) (Table 2). PD-L1 expression in tumour cells did not significantly associate with survival (Supplementary Table S5). We further evaluated the prognostic significance of PD-L1^+^ and PD-L1^−^ macrophage densities in relation to MMR status and stage. The survival associations of PD-L1^+^ or PD-L1^−^ macrophage densities did not significantly differ by MMR status (Supplementary Table S6) or stage (Supplementary Tables S7, S8) in univariable or multivariable analysis (all *P*_interaction_ > 0.005).

**Table 2:** Univariable and multivariable Cox regression models for colorectal cancer-specific and overall survival according to the total, PD-L1^+^, and PD-L1^−^ macrophage densities in the tumour centre and the invasive margin.

	No. of cases	Colorectal cancer-specific survival			Overall survival		
		No. of events	Univariable HR (95% CI)	Multivariable HR (95% CI)	No. of events	Univariable HR (95% CI)	Multivariable HR (95% CI)
Tumour centre							
Macrophage density							
Q1	228	64	1 (referent)	1 (referent)	116	1 (referent)	1 (referent)
Q2	228	56	0.85 (0.59–1.21)	0.81 (0.56–1.18)	109	0.93 (0.71–1.21)	0.92 (0.70–1.20)
Q3	227	63	0.97 (0.69–1.38)	0.84 (0.59–1.20)	107	0.93 (0.72–1.21)	0.84 (0.64–1.10)
Q4	227	59	0.93 (0.65–1.32)	0.80 (0.56–1.16)	117	1.03 (0.80–1.33)	0.84 (0.65–1.10)
* P*_trend_			0.87	0.29		0.81	0.17
PD-L1^+^ macrophage density							
Q1	228	83	1 (referent)	1 (referent)	134	1 (referent)	1 (referent)
Q2	228	61	0.68 (0.49–0.94)	0.81 (0.57–1.13)	106	0.72 (0.56–0.93)	0.82 (0.64–1.07)
Q3	227	54	0.59 (0.42–0.83)	0.75 (0.53–1.06)	102	0.68 (0.53–0.88)	0.73 (0.56–0.95)
Q4	227	44	0.47 (0.33–0.69)	0.63 (0.43–0.93)	107	0.70 (0.54–0.90)	0.72 (0.55–0.94)
* P*_trend_			<0.0001	0.015		0.0050	0.011
PD-L1^–^ macrophage density							
Q1	228	59	1 (referent)	1 (referent)	110	1 (referent)	1 (referent)
Q2	228	52	0.86 (0.59–1.25)	0.75 (0.51–1.09)	111	1.01 (0.77–1.31)	0.93 (0.71–1.22)
Q3	227	58	0.97 (0.68–1.39)	0.88 (0.61–1.27)	108	0.99 (0.73–1.29)	0.93 (0.71–1.21)
Q4	227	73	1.31 (0.93–1.85)	0.85 (0.60–1.21)	120	1.18 (0.91–1.53)	0.88 (0.67–1.14)
* P*_trend_			0.085	0.62		0.25	0.35
Invasive margin							
Macrophage density							
Q1	228	67	1 (referent)	1 (referent)	113	1 (referent)	1 (referent)
Q2	228	62	0.94 (0.66–1.32)	1.16 (0.81–1.66)	118	1.07 (0.83–1.39)	1.19 (0.91–1.56)
Q3	227	47	0.67 (0.46–0.97)	0.92 (0.62–1.36)	100	0.84 (0.64–1.09)	0.98 (0.74–1.28)
Q4	227	66	0.99 (0.70–1.39)	0.93 (0.66–1.32)	118	1.05 (0.81–1.36)	0.98 (0.76–1.28)
* P*_trend_			0.54	0.46		0.83	0.57
PD-L1^+^ macrophage density							
Q1	228	92	1 (referent)	1 (referent)	136	1 (referent)	1 (referent)
Q2	228	71	0.75 (0.55–1.02)	1.07 (0.78–1.48)	116	0.82 (0.64–1.06)	1.02 (0.79–1.32)
Q3	227	43	0.43 (0.30–0.61)	0.68 (0.47–0.99)	97	0.64 (0.50–0.83)	0.85 (0.65–1.12)
Q4	227	36	0.34 (0.23–0.50)	0.52 (0.34–0.78)	100	0.62 (0.48–0.80)	0.69 (0.52–0.91)
* P*_trend_			<0.0001	0.0004		<0.0001	0.0053
PD-L1^–^ macrophage density							
Q1	228	55	1 (referent)	1 (referent)	113	1 (referent)	1 (referent)
Q2	228	58	1.08 (0.74–1.56)	1.33 (0.91–1.94)	108	0.98 (0.75–1.28)	1.08 (0.83–1.42)
Q3	227	50	0.88 (0.60–1.29)	1.02 (0.69–1.50)	101	0.86 (0.66–1.13)	0.95 (0.72–1.25)
Q4	227	79	1.53 (1.08–2.16)	1.36 (0.96–1.92)	127	1.21 (0.94–1.56)	1.13 (0.88–1.46)
* P*_trend_			0.038	0.21		0.27	0.52

The densities were divided into ordinal quartile categories from low (Q1) to high (Q4).

Multivariable Cox proportional hazards regression models were adjusted for sex (male, female), age (<65, 65–75, >75), year of operation (2000–2005, 2006–2010, 2011–2015), tumour location (proximal colon, distal colon, rectum), stage (I–II, III, IV), tumour grade (well/moderately differentiated, poorly differentiated), lymphovascular invasion (negative, positive), MMR status (proficient, deficient), and *BRAF* status (wild-type, mutant).

*P*_trend_ values were calculated by using the four categories of immune cell densities as continuous variables in univariable and multivariable Cox proportional hazard regression models.

*CI* confidence interval, *HR* hazard ratio.

Higher T cell density associated with better cancer-specific survival regardless of PD-1 expression in both univariable (Fig. 2b, Table 3) and multivariable analyses (Table 3). In multivariable analyses, the HR for high PD-1^+^ T cell density in the tumour centre (Q4 vs. Q1) was 0.46 (95% CI: 0.30–0.71), while the HR for high PD-1^−^ T cell density (Q4 vs. Q1) was 0.48 (95% CI: 0.32–0.70) and the HR for high T cell density (Q4 vs. Q1) was 0.49 (95% CI: 0.33–0.73). Full multivariable Cox regression models with all variables are shown in Supplementary Table S9.

**Table 3:** Univariable and multivariable Cox regression models for cancer-specific and overall survival according to the densities of total, PD-1^+^, and PD-1^–^ T cells in the tumour centre and the invasive margin.

	No. of cases	Colorectal cancer-specific survival			Overall survival		
		No. of events	Univariable HR (95% CI)	Multivariable HR (95% CI)	No. of events	Univariable HR (95% CI)	Multivariable HR (95% CI)
Tumour centre							
T cell density							
Q1	228	88	1 (referent)	1 (referent)	146	1 (referent)	1 (referent)
Q2	228	58	0.55 (0.40–0.77)	0.72 (0.51–1.01)	103	0.58 (0.45–0.74)	0.68 (0.53–0.88)
Q3	227	56	0.54 (0.39–0.76)	0.67 (0.47–0.94)	100	0.57 (0.44–0.74)	0.62 (0.48–0.80)
Q4	227	40	0.38 (0.26–0.55)	0.49 (0.33–0.73)	100	0.56 (0.43–0.72)	0.59 (0.45–0.77)
* P*_trend_			<0.0001	0.0003		<0.0001	<0.0001
PD-1^+^ T cell density							
Q1	228	91	1 (referent)	1 (referent)	147	1 (referent)	1 (referent)
Q2	228	57	0.55 (0.39–0.76)	0.82 (0.58–1.16)	103	0.60 (0.47–0.77)	0.78 (0.60–1.01)
Q3	227	63	0.59 (0.43–0.82)	0.75 (0.54–1.04)	109	0.63 (0.49–0.81)	0.68 (0.53–0.87)
Q4	227	31	0.28 (0.18–0.42)	0.46 (0.30–0.71)	90	0.49 (0.38–0.64)	0.57 (0.43–0.76)
* P*_trend_			<0.0001	0.0006		<0.0001	<0.0001
PD-1^–^ T cell density							
Q1	228	86	1 (referent)	1 (referent)	145	1 (referent)	1 (referent)
Q2	228	55	0.54 (0.38–0.76)	0.68 (0.48–0.96)	103	0.58 (0.45–0.75)	0.69 (0.54–0.90)
Q3	227	61	0.61 (0.44–0.85)	0.67 (0.48–0.94)	106	0.63 (0.49–0.81)	0.61 (0.47–0.79)
Q4	227	40	0.39 (0.27–0.57)	0.48 (0.32–0.70)	95	0.54 (0.42–0.70)	0.55 (0.42–0.71)
* P*_trend_			<0.0001	0.0002		<0.0001	<0.0001
Invasive margin							
T cell density							
Q1	228	94	1 (referent)	1 (referent)	146	1 (referent)	1 (referent)
Q2	228	67	0.66 (0.48–0.90)	0.83 (0.60–1.14)	103	0.71 (0.56–0.91)	0.80 (0.62–1.03)
Q3	227	38	0.34 (0.23–0.49)	0.53 (0.36–0.78)	100	0.51 (0.39–0.66)	0.65 (0.50–0.86)
Q4	227	43	0.39 (0.27–0.56)	0.58 (0.40–0.85)	100	0.58 (0.45–0.75)	0.64 (0.48–0.83)
* P*_trend_			<0.0001	0.0005		<0.0001	0.0003
PD-1^+^ T cell density							
Q1	228	97	1 (referent)	1 (referent)	141	1 (referent)	1 (referent)
Q2	228	57	0.52 (0.38–0.72)	0.81 (0.58–1.14)	112	0.71 (0.55–0.90)	0.90 (0.70–1.17)
Q3	227	55	0.49 (0.35–0.68)	0.83 (0.58–1.17)	101	0.61 (0.47–0.79)	0.82 (0.63–1.08)
Q4	227	33	0.29 (0.19–0.43)	0.52 (0.34–0.79)	95	0.56 (0.43–0.72)	0.70 (0.53–0.93)
* P*_trend_			<0.0001	0.0043		<0.0001	0.011
PD-1^–^ T cell density							
Q1	228	94	1 (referent)	1 (referent)	144	1 (referent)	1 (referent)
Q2	228	60	0.60 (0.43–0.82)	0.80 (0.58–1.12)	105	0.66 (0.51–0.85)	0.77 (0.59–0.99)
Q3	227	43	0.40 (0.28–0.58)	0.62 (0.43–0.90)	101	0.60 (0.46–0.77)	0.75 (0.57–0.97)
Q4	227	45	0.42 (0.29–0.60)	0.61 (0.42–0.88)	99	0.58 (0.45–0.76)	0.65 (0.50–0.85)
* P*_trend_			<0.0001	0.0027		<0.0001	0.0021

The densities were divided into ordinal quartile categories from low (Q1) to high (Q4).

Multivariable Cox proportional hazards regression models were adjusted for sex (male, female), age (<65, 65–75, >75), year of operation (2000–2005, 2006–2010, 2011–2015), tumour location (proximal colon, distal colon, rectum), stage (I–II, III, IV), tumour grade (well/moderately differentiated, poorly differentiated), lymphovascular invasion (negative, positive), MMR status (proficient, deficient), and *BRAF* status (wild-type, mutant).

*P*_trend_ values were calculated by using the four categories of immune cell densities as continuous variables in univariable and multivariable Cox proportional hazard regression models.

*CI* confidence interval, *HR* hazard ratio.

Considering potential interactions between PD-1 and PD-L1, we next evaluated the prognostic value of PD-1^+^ T cell density in tumour groups defined by PD-L1^+^ macrophage densities or tumour cell PD-L1 expression. These analyses indicated that the survival associations of PD-1^+^ T cells did not statistically significantly differ according to the density of PD-L1^+^ macrophages or PD-L1 expression in tumour cells in univariable (Supplementary Fig. S11, Supplementary Tables S10, S11) or multivariable analyses (Supplementary Tables S10, S11) (all *P*_interaction_ > 0.005).

In secondary analyses, we investigated the prognostic significance of M1-like and M2-like macrophage densities. Higher M1-like macrophage density associated with longer cancer-specific survival in both the tumour centre and the invasive margin in univariable analyses (Supplementary Fig. S9, Supplementary Table S12). The prognostic value remained significant in the tumour centre in multivariable analyses [*P*_trend_ = 0.0004, HR for high (Q4 vs. Q1) 0.54, 95% CI: 0.38–0.78] (Supplementary Table S12). Higher M2-like macrophage density in the tumour centre associated with longer cancer-specific survival and higher M1:M2-like macrophage density ratio in the tumour centre and the invasive margin associated with worse cancer-specific survival in univariable analyses (Supplementary Fig. S9, Supplementary Table S12) but did not reach statistical significance in multivariable analysis (*P*_trend_ > 0.005). To evaluate the prognostic value of PD-L1 expression in differently polarised macrophage subpopulations, we calculated macrophage densities based on subpopulations defined by both PD-L1 expression and polarisation state (Supplementary Table S13). We found that higher PD-L1^+^ M1-like macrophage density in both the tumour centre and the invasive margin predicted longer cancer-specific survival in univariable and multivariable models (multivariable *P*_trend_ = 0.0005 and *P*_trend_ = 0.0008, respectively), while the densities of PD-L1^−^ M1-like macrophages, PD-L1^+^ M2-like macrophages, or PD-L1^−^ M2-like macrophages did not significantly associate with the survival in multivariable models (all *P*_trend_ > 0.005). These results indicate that higher densities of PD-L1^+^ and M1-like macrophages associate with favourable prognosis, which is further highlighted when PD-L1 expression and polarisation are examined simultaneously.

### Spatial analyses

**Fig. 3:**
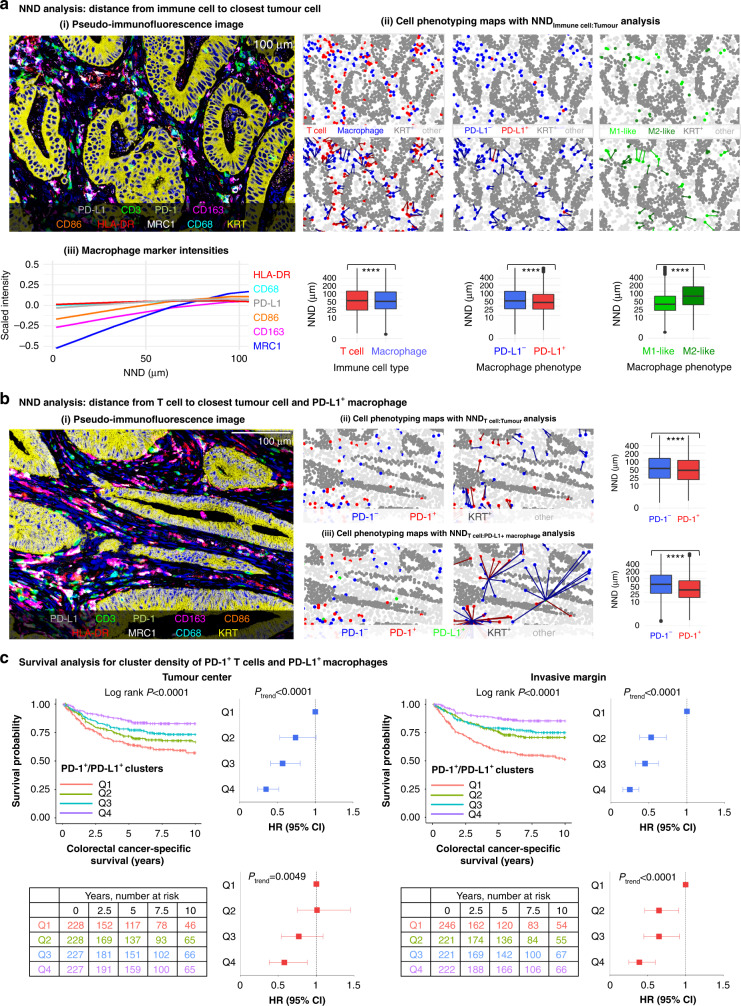
Spatial analysis of immune cells using the nearest neighbour distance (NND) function and the cancer-specific survival analysis for PD-1/PD-L1 cluster density. **a** pseudo-immunofluorescence image from a tumour site showing all nine markers (i). Cell phenotyping maps with nearest neighbour distance analysis from each immune cell to the closest tumour cell and boxplots visualising the distribution of nearest neighbour distances across all tumour images (*N* = 3,190). The statistical significance was tested with Wilcoxon rank-sum test. ****P < 0.0001 (ii). The expression levels of various phenotypic markers in macrophages according to the distance to the closest tumour cell. The plots are based on 1,582,095 T cells and 1,788,538 macrophages (iii). **b** pseudo-immunofluorescence image from a tumour site showing all nine markers (i). Cell phenotyping maps and nearest neighbour distance analyses from each PD-1^+^ and PD-1^–^ T cell to the closest tumour cell (ii) and to the closest PD-L1^+^ macrophage (iii). Boxplots visualise the distribution of nearest neighbour distances across all tumour images (*N* = 3,190). The significance was tested with Wilcoxon rank-sum test. ****P < 0.0001. **c** Kaplan–Meier survival curves and Cox proportion hazards regression models for cancer-specific survival for PD-1^+^ T cell/PD-L1^+^ macrophage cluster densities in 910 patients in the tumour centre and the invasive margin. One cluster is composed of one PD-1^+^ T cell with at least one PD-L1^+^ macrophage within a 20 µm radius. The cluster densities were divided into ordinal quartiles from low (Q1) to high (Q4). Statistical significance for Kaplan–Meier survival estimates were determined with Log-rank test. Univariable (blue) and multivariable (red) Cox proportional hazards regression models are represented as forest plots with HRs along with their 95% CIs as whiskers. Multivariable Cox proportional hazards regression models were adjusted for sex (male, female), age (<65, 65–75, >75), year of operation (2000–2005, 2006–2010, 2011–2015), tumour location (proximal colon, distal colon, rectum), stage (I–II, III, IV), tumour grade (well/moderately differentiated, poorly differentiated), lymphovascular invasion (negative, positive), MMR status (proficient, deficient), and *BRAF* status (wild-type, mutant).

We characterised the spatial arrangement of immune cells by measuring the average distances from immune cells to the nearest tumour cell with NND analysis. Macrophages were located 4.6% closer to tumour cells than T cells on average. Of macrophages, PD-L1^+^ macrophages were located 24% closer to tumour cells than PD-L1^−^ macrophages (*P* < 0.0001) and M1-like macrophages were 48% closer to tumour cells than M2-like macrophages (*P* < 0.0001) (Fig. 3a). To further visualise these findings, we plotted scaled intensities of macrophage polarisation markers and PD-L1 as a function of NND from tumour cells using generalised additive model smoothing (Fig. 3a). This plot showed that M2-like macrophage markers (MRC1 and CD163) had lower scaled intensities at tumour cell proximity than other macrophage markers. The Kaplan–Meier curves showed that the spatial proximity of macrophages (total, PD-L1^+^, or PD-L1^−^) or T cells (total, PD-1^+^, or PD-1^−^) with tumour cells, as measured with mean NNDs from immune cell to the closest tumour cell, did not significantly associate with cancer-specific survival (*P* > 0.005) (Supplementary Fig. S12).

For T cells, we measured NNDs to the closest tumour cell and to the closest PD-L1^+^ macrophage to evaluate the possibility for PD-1*−*PD-L1 interactions. We found that PD-1^+^ T cells were located closer to both tumour cells (difference 13%, *P* < 0.0001) and PD-L1^+^ macrophages (difference 35%, *P* < 0.0001) than PD-1^−^ T cells (Fig. 3b). To evaluate the prognostic value of PD-1^+^ T cells co-localised with PD-L1^+^ macrophages, we calculated the density of PD-1^+^/PD-L1^+^ clusters. The mean cluster densities in the tumour centre and the invasive margin were 34 clusters/mm^2^ and 47 clusters/mm^2^, respectively. Higher density of PD-1^+^/PD-L1^+^ clusters associated with longer cancer-specific survival in both the tumour centre and the invasive margin in univariable and multivariable analyses (Fig. 3c). The prognostic value was slightly stronger in the invasive margin, in which the multivariable HR for high cluster density (Q4 vs. Q1) was 0.39 (95% CI 0.25–0.60, *P*_trend_ < 0.0001).

## Discussion

In the present study, we used multiplex immunohistochemistry combined with digital image analysis and quantitative density and spatial analysis to comprehensively characterise the expression of PD-L1 (CD274) and PD-1 (PDCD1) immune checkpoints in the colorectal cancer microenvironment. This analysis, conducted in a large, population-based cohort of 910 colorectal cancer patients expands the knowledge on immune checkpoint expression patterns, their prognostic value, and associations with T cell and macrophage infiltration and may help to develop cancer therapies.

The expression of PD-L1 and PD-1 is often elevated in cancer and their interaction may repress the cytotoxic activity of T cells and thus promote tumour immune escape [4]. However, the prognostic value of PD-L1 is incompletely established in colorectal cancer and prior studies have reached contradictory conclusions [20]. We found that PD-L1 expression was more frequently present in macrophages than in tumour cells. Although PD-L1 is commonly thought to have immunosuppressive effect, we found that higher density of PD-L1^+^ macrophages in the invasive margin associated with longer colorectal cancer-specific survival independent of stage, grade, and MMR status. Our finding is concordant with some prior studies which have reported association between high PD-L1 expression in immune cells and better survival [21–23] and adds to these findings by more accurately defining phenotypes of the immune cells expressing PD-L1. In our study, PD-L1 expression in tumour cells did not associate with prognosis. Prior colorectal cancer studies have reported inconsistent deductions of the prognostic value of PD-L1^+^ expression in tumour cells [22–27]. Divergent results may be due to the small percentage of PD-L1 positive tumours and variability in antibody selection, quantification methods, or setting cut-off value for the positivity.

The detection and phenotyping of macrophages are challenging because of their plasticity and the lack of specific or standardised immunohistochemical markers for various subpopulations [7]. We used multiplex immunohistochemistry instead of conventional single-plex chromogenic staining, enabling us to phenotype cells with multimarker combinations and to analyse spatial relationships between cells. To separate M1-like and M2-like polarisation states, we utilised two markers for both phenotypes, in line with two previous studies [16, 28], and only included the extremes of the polarisation spectrum in our main analyses. We found that higher density of M1-like macrophages in the tumour centre associated with longer cancer-specific survival, which is in line with some previous studies [29–31], while M2-like macrophages tended to associate with shorter survival, but not significantly in multivariable models.

In analyses combining PD-L1 expression and polarisation status of macrophages, PD-L1^+^ macrophages were more likely M1-polarised than M2-polarised, which could partly explain their comparable spatial arrangement and prognostic value. The associations of macrophage polarisation phenotypes with PD-L1 expression are not well-characterised, but may be affected by the cytokine environment [32]. In particular, IFNG can induce M1-like macrophage polarisation and PD-L1 expression in macrophages [33, 34]. However, other studies have found that certain cytokines, such as IL6 and IL10, increase PD-L1 expression but drive macrophage polarisation towards an M2-phenotype [33, 35, 36]. Our analyses suggested that the prognostic value of macrophages was influenced by both the polarisation phenotype (M1-like vs. M2-like) and PD-L1 expression, with PD-L1^+^ M1-like phenotype showing the strongest association with a favourable outcome.

Some recent studies have reported the co-localisation of immune cells with tumour cells to be strongly prognostic [16, 37–39]. Interestingly, macrophage subtypes frequently located in closer proximity to tumour cells (PD-L1^+^ and M1-like macrophages) also showed the strongest associations with favourable clinical outcome. Shorter average distance of M1-like than M2-like macrophages from the closest tumour cell has been reported also in prior studies of gastric [28], lung [29] and pancreatic [16] cancers. Based on these findings, we hypothesise that the greater co-localisation between macrophages and tumour cells could increase the probability of cell contacts, thus allowing enhanced anti-tumoral macrophage function.

Our study addressed the prognostic significance of T cell subsets defined by PD-1 expression. We found that higher densities of both PD-1^+^ and PD-1^−^ T cells showed strong associations with favourable survival, and their HR point estimates were very close to that of the overall T cell population. This supports the strong prognostic significance of T cells in general [40], and indicates that it appears to be independent of PD-1 expression by T cells. Our findings were in line with some prior studies in colorectal cancer that have reported associations between higher PD-1^+^ cell densities and longer survival [22, 24, 25].

To evaluate the possibility of PD-1/PD-L1 interactions occurring in the tumour microenvironment, we assessed the correlations between the densities of immune checkpoint expressing (PD-1^+^ and PD-L1^+^) cells, as well as their co-localisation. In line with prior reports [21, 22, 24], we found a moderate correlation between PD-L1^+^ macrophage and T cell densities. Furthermore, this correlation was higher for PD-1^+^ T cells than for PD-1^−^ T cells. A prior study [25] reported that mismatch repair deficient colorectal tumours with high PD-1 expression associated with worse recurrence-free survival if PD-L1 expression was high, while high expression of PD-1 associated with prolonged survival if PD-L1 expression was low. In our cohort, such findings were not identified, as higher PD-1^+^ T cell densities associated with favourable outcome regardless of PD-L1 expression in tumour cells or PD-L1^+^ macrophage densities. Furthermore, higher density of PD-1^+^ T cell/PD-L1^+^ macrophage clusters strongly associated with longer cancer-specific survival.

In contrast to our hypotheses, higher densities of PD-L1^+^ macrophages and PD-1^+^ T cells individually, as well as higher density of PD-1^+^ T cell/PD-L1^+^ macrophage clusters associated with favourable prognostic impact. It has been well known that certain cancer risk factors (such as obesity and Lynch syndrome genetic mutations) may associate with better clinical outcomes among patients with a given cancer type. Those apparent paradoxical findings can be explained by interpersonal heterogeneity of cancer [41]. Our findings may be explained by the strong induction of these immune checkpoints as a compensatory response to generally elevated anti-tumour inflammatory reaction in immunologically hot tumours [22, 38]. Favourable outcome of high immune checkpoint expression could also be related to IFNG, which is secreted mainly by activated infiltrating T cells and NK cells. High IFNG expression is correlated with both PD-L1 and PD-1 expression and associates with better colorectal cancer prognosis [24]. Furthermore, gut microbiota e.g., *Fusobacterium nucleatum*, might associate with T cell count, PD-1 and PD-L1 expression, and colorectal cancer prognosis [42].

To our knowledge, this is the first study examining the PD-L1/PD-1 expression along with macrophage polarisation using multimarker analysis. However, the findings of this study should be interpreted with some caution. First, the analyses were conducted using tissue microarrays, which may not fully represent the immune infiltration in the whole tumour area [43]. To increase the validity of our results, we analysed multiple cores from each tumour (average: 3.5 cores) and the cores were selected from different sites representing average immune cell infiltrates. Reasonably good core-to-core correlations indicated that the number of analysed tissue microarray cores was adequate. By using tissue microarrays, we could examine 910 tumours cost-efficiently and select only representative tumour areas to be stained and analysed. Second, PD-L1 staining patterns may differ between antibody clones. We decided to use a well-validated clone (E1L3N) that is frequently applied for clinical use in evaluating PD-L1 status in lung cancer [44]. However, the optimal antibody for colorectal cancer is yet to be determined. Third, macrophage phenotyping requires several markers and there are no single consensus markers for M1/M2 polarisation states. We used two polarisation markers for both M1 and M2 subpopulations and determined the polarisation states in line with prior reports [16, 45]. Fourth, the cell detection algorithm of QuPath (image analysis software that was utilised) is not able to segment cell membrane, and we used cytoplasmic staining intensities for markers that are expressed in either cell cytoplasm or cell membrane. Fifth, most of the patients were non-Hispanic white, and we excluded patients with preoperative treatment, which led to underrepresentation of rectal cancers. Therefore, the applicability of the results for patients of different ethnicities and with preoperative treatments needs to be confirmed by independent studies. Furthermore, the patients were operated along a period of 16 years, during which the cancer treatments have developed. To mitigate possible bias related to this, we included year of operation as a covariate in multivariable survival models. Sixth, the information on extramural venous invasion and perineural invasion were not available, although they are strong prognostic indicators in colorectal cancer. Seventh, although multiplex immunohistochemistry analysis enabled detailed immune cell characterisation, the method is laborious and would need further automation and validation to be applied as a biomarker in clinical setting.

This study has important strengths. Our study included a large, comprehensively analysed [9, 22, 46–49] population-based cohort evaluated in accordance with the latest guidelines. All tumours were also screened for MMR status and *BRAF* mutation status representing two key molecular features of colorectal cancer. Multiplex immunohistochemistry staining together with machine learning-based image analysis enabled accurate phenotyping of each cell with multimarker combination in a single batch, facilitating the consistency and reproducibility of the analysis. Flow cytometry and RNA sequencing methods are other commonly used myeloid cell phenotyping methods but, unlike multiplex immunohistochemistry, they do not provide the spatial information of the cells.

In conclusion, this study shows that higher density of PD-L1 expressing macrophages and their spatial proximity with PD-1 expressing T cells associate with prolonged survival of colorectal cancer patients. Our results highlight the utility of detailed multimarker analysis in understanding the role of PD-L1 and PD-1 expression in cancer immune escape and developing improved immunotherapies.

## Supplementary information

Supplementary material

## Data Availability

The datasets generated and/or analysed during this study are not publicly available. The sharing of data will require approval from relevant ethics committees and/or biobanks. Further information including the procedures to obtain and access data of Finnish Biobanks are described at https://finbb.fi/en/fingenious-service.
