# Supplementary material for: Spatially resolved multimarker evaluation of CD274 (PD-L1)/PDCD1 (PD-1) immune checkpoint expression and macrophage polarisation in colorectal cancer
Source: Br J Cancer. 2023 Mar 31;128(11):2104–15. doi: 10.1038/s41416-023-02238-6 (PMC10205752; doi:10.1038/s41416-023-02238-6)
Supplement: Supplementary file 1 — Supplementary material [file 41416_2023_2238_MOESM1_ESM.pdf]

Supplementary tables

Table S1 Antibodies and multiplex immunohistochemistry protocols in the staining order.

Marker	Clone	Manufacturer	Catalog number	Host species	Antigen retrieval	Antibody dilution, incubation time
PD-L1	E1L3N	Cell Signaling	13684S	rabbit	ER2, 30 min	1:100, 30 min
CD3	LN10	Leica	PA055	mouse	ER1, 60 min	ready-to-use, 30 min
PD-1	SP269	Abcam	ab227681	rabbit	ER1, 30 min	1:40, 60 min
CD163	10D6	Thermo Scientific	MS-1103	mouse	ER1, 60 min	1:100, 30 min
CD86	E2G8P	Cell Signaling	91882S	rabbit	ER1, 30 min	1:50, 30 min
HLA-DR	TAL 1B5	Santa Cruz	sc-53319	mouse	ER1, 60 min	1:4000, 30 min
MRC1	E2L9N	Cell Signaling	91992S	rabbit	ER1, 30 min	1:500, 30 min
CD68	KP1	Biolegend	916104	mouse	ER1, 30 min	1:40 000, 30 min
KRT	BS5	BioSite Histo	BSH-7124-1	mouse	ER1, 60 min	1:400, 30 min

Table S2 Demographic and clinical characteristics of colorectal cancer cases according to PD-L1 expression in tumor cells.

Characteristic	Total N	PD-L1 expression in tumor cells positive	PD-L1 expression in tumor cells negative	*P*
All cases	910 (100%)	66 (7%)	844 (93%)	
Sex				0.16
Male	462 (51%)	28 (42%)	434 (51%)	
Female	448 (49%)	38 (58%)	410 (49%)	
Tumor location				<0.0001
Proximal colon	445 (49%)	53 (80%)	392 (46%)	
Distal colon	332 (36%)	8 (12%)	324 (38%)	
Rectum	133 (15%)	5 (7.6%)	128 (15%)	
AJCC stage				0.011
I	151 (17%)	6 (9.1%)	145 (17%)	
II	342 (38%)	37 (56%)	305 (36%)	
III	301 (33%)	18 (27%)	283 (34%)	
IV	116 (13%)	5 (7.6%)	111 (13%)	
Tumor grade				<0.0001
Low-grade (well to moderately differentiated)	760 (84%)	38 (58%)	722 (86%)	
High-grade (poorly differentiated)	150 (16%)	28 (42%)	122 (15%)	
Lymphovascular invasion				0.37
No	719 (79%)	55 (83%)	664 (79%)	
Yes	191 (21%)	11 (17%)	180 (21%)	
MMR status				<0.0001
MMR proficient	773 (85%)	36 (55%)	737 (87%)	
MMR deficient	137 (15%)	30 (46%)	107 (13%)	
BRAF status				<0.0001
Wild-type	763 (84%)	37 (56%)	726 (86%)	
Mutant	147 (16%)	29 (44%)	118 (14%)	

Tumors were categorized into PD-L1 negative or positive using histoscore method with a cut-off >5 for the positivity.
Abbreviations: AJCC, American Joint Committee on Cancer; MMR, mismatch repair.

Table S3 Multivariable Cox regression models for cancer-specific survival according to PD-L1⁺ and PD-L1⁻ macrophage densities in the tumor center and the invasive margin with all variables.

	**PD-L1⁺ macrophage density**		**PD-L1⁻ macrophage density**	
	Tumor center	Invasive margin	Tumor center	Invasive margin
	HR (95% CI)	HR (95% CI)	HR (95% CI)	HR (95% CI)
PD-L1⁺ macrophage density			Not included	Not included
Q1	1 (referent)	1 (referent)		
Q2	0.81 (0.57-1.13)	1.07 (0.78-1.48)		
Q3	0.75 (0.53-1.06)	0.68 (0.47-0.99)		
Q4	0.63 (0.43-0.93)	0.52 (0.34-0.78)		
PD-L1⁻ macrophage density	Not included	Not included		
Q1			1 (referent)	1 (referent)
Q2			0.75 (0.51-1.09)	1.33 (0.91-1.94)
Q3			0.88 (0.61-1.27)	1.02 (0.69-1.50)
Q4			0.85 (0.60-1.21)	1.36 (0.96-1.92)
Age				
<65	1 (referent)	1 (referent)	1 (referent)	1 (referent)
65–75	1.18 (0.86-1.64)	1.14 (0.83-1.48)	1.21 (0.87-1.67)	1.15 (0.83-1.59)
>75	1.96 (1.42-2.72)	1.96 (1.41-2.71)	1.95 (1.41-2.71)	1.90 (1.37-2.63)
Sex				
Male	1 (referent)	1 (referent)	1 (referent)	1 (referent)
Female	0.87 (0.67-1.14)	0.85 (0.65-1.11)	0.87 (0.67-1.14)	0.87 (0.67-1.14)
Year of operation				
2000–2005	1 (referent)	1 (referent)	1 (referent)	1 (referent)
2006–2010	0.60 (0.44-0.82)	0.59 (0.43-0.81)	0.59 (0.43-0.81)	0.61 (0.44-0.83)
2011–2015	0.44 (0.32-0.61)	0.43 (0.31-0.59)	0.43 (0.31-0.60)	0.44 (0.32-0.61)
Tumor location				
Proximal colon	1 (referent)	1 (referent)	1 (referent)	1 (referent)
Distal colon	0.95 (0.71-1.27)	0.95 (0.72-1.27)	0.98 (0.73-1.30)	0.94 (0.70-1.26)
Rectum	0.81 (0.54-1.23)	0.79 (0.52-1.20)	0.80 (0.53-1.21)	0.78 (0.52-1.18)
AJCC stage				
I–II	1 (referent)	1 (referent)	1 (referent)	1 (referent)
III	2.76 (1.95-3.92)	2.68 (1.89-3.81)	2.88 (2.03-4.08)	2.88 (2.04-4.09)
IV	16.9 (11.7-24.5)	16.4 (11.3-23.9)	17.6 (12.2-25.6)	17.5 (12.0-25.5)
Tumor grade				
Low-grade (well to moderately differentiated)	1 (referent)	1 (referent)	1 (referent)	1 (referent)
High-grade (poorly differentiated)	1.91 (1.35-2.70)	1.98 (1.40-2.81)	1.81 (1.28-2.57)	1.82 (1.29-2.56)
Lymphovascular invasion				
No	1 (referent)	1 (referent)	1 (referent)	1 (referent)
Yes	2.05 (1.55-2.70)	1.98 (1.50-2.61)	2.13 (1.61-2.81)	2.06 (1.56-2.72)
MMR status				
MMR proficient	1 (referent)	1 (referent)	1 (referent)	1 (referent)
MMR deficient	0.57 (0.32-1.01)	0.63 (0.35-1.13)	0.53 (0.30-0.94)	0.51 (0.29-0.91)
BRAF mutation				
Wild-type	1 (referent)	1 (referent)	1 (referent)	1 (referent)
Mutant	1.33 (0.84-2.12)	1.34 (0.84-2.13)	1.33 (0.83-2.12)	1.32 (0.82-2.12)

The densities were divided into ordinal quartile categories from low (Q1) to high (Q4).
Abbreviations: AJCC, American Joint Committee on Cancer; CI, confidence interval; HR, hazard ratio; MMR, mismatch repair.

Table S4 Univariable and multivariable Cox regression models for cancer-specific and overall survival according to PD-L1⁺ and PD-L1⁻ macrophage densities in tumor intraepithelial and stromal regions of the tumor center and the invasive margin.

		Colorectal cancer-specific survival			Overall survival		
	No. of cases	No. of events	Univariable HR (95% CI)	Multivariable HR (95% CI)	No. of events	Univariable HR (95% CI)	Multivariable HR (95% CI)
**Tumor center**							
Intraepithelial PD-L1⁺ macrophage density							
Q1	228	80	1 (referent)	1 (referent)	126	1 (referent)	1 (referent)
Q2	228	62	0.72 (0.52-1.00)	0.85 (0.61-1.18)	109	0.79 (0.61-1.01)	0.86 (0.67-1.12)
Q3	227	49	0.56 (0.39-0.80)	0.66 (0.46-0.94)	106	0.75 (0.58-0.97)	0.78 (0.60-1.01)
Q4	227	51	0.60 (0.42-0.85)	0.81 (0.55-1.20)	108	0.78 (0.61-1.01)	0.75 (0.56-1.00)
P_{trend}			0.0012	0.089		0.059	0.030
Stromal PD-L1⁺ macrophage density							
Q1	228	83	1 (referent)	1 (referent)	134	1 (referent)	1 (referent)
Q2	228	63	0.68 (0.49-0.94)	0.88 (0.63-1.22)	104	0.69 (0.54-0.90)	0.82 (0.63-1.07)
Q3	227	56	0.60 (0.43-0.84)	0.78 (0.55-1.11)	107	0.69 (0.54-0.89)	0.76 (0.58-0.99)
Q4	227	40	0.41 (0.28-0.60)	0.61 (0.41-0.91)	104	0.66 (0.51-0.85)	0.71 (0.54-0.94)
P_{trend}			<0.0001	0.013		0.0019	0.012
Intraepithelial PD-L1⁻ macrophage density							
Q1	228	59	1 (referent)	1 (referent)	110	1 (referent)	1 (referent)
Q2	228	64	1.11 (0.78-1.58)	1.14 (0.80-1.64)	112	1.03 (0.79-1.34)	1.03 (0.79-1.35)
Q3	227	58	1.04 (0.72-1.49)	0.88 (0.61-1.28)	113	1.10 (0.85-1.43)	0.95 (0.73-1.25)
Q4	227	61	1.11 (0.77-1.58)	0.95 (0.65-1.41)	114	1.10 (0.85-1.43)	0.84 (0.63-1.11)
P_{trend}			0.68	0.51		0.40	0.19
Stromal PD-L1⁻ macrophage density							
Q1	228	54	1 (referent)	1 (referent)	108	1 (referent)	1 (referent)
Q2	228	64	1.19 (0.83-1.70)	0.96 (0.66-1.40)	110	1.03 (0.79-1.35)	0.94 (0.71-1.23)
Q3	227	56	1.04 (0.72-1.51)	0.91 (0.63-1.34)	109	1.02 (0.78-1.33)	0.93 (0.71-1.22)
Q4	227	68	1.33 (0.93-1.90)	0.97 (0.67-1.39)	122	1.21 (0.94-1.57)	0.98 (0.76-1.28)
P_{trend}			0.21	0.81		0.18	0.91
**Invasive margin**							
Intraepithelial PD-L1⁺ macrophage density							
Q1	233	84	1 (referent)	1 (referent)	126	1 (referent)	1 (referent)
Q2	226	70	0.86 (0.63-1.18)	1.04 (0.75-1.44)	120	1.00 (0.78-1.28)	1.12 (0.87-1.45)
Q3	225	53	0.62 (0.44-0.88)	0.85 (0.59-1.20)	103	0.81 (0.63-1.05)	0.96 (0.73-1.25)
Q4	226	35	0.39 (0.26-0.58)	0.51 (0.33-0.78)	100	0.73 (0.56-0.95)	0.75 (0.56-1.00)
P_{trend}			<0.0001	0.0030		0.0073	0.044
Stromal PD-L1⁺ macrophage density							
Q1	228	95	1 (referent)	1 (referent)	139	1 (referent)	1 (referent)
Q2	228	68	0.68 (0.50-0.93)	0.98 (0.71-1.36)	116	0.79 (0.61-1.00)	1.06 (0.82-1.36)
Q3	227	49	0.45 (0.32-0.64)	0.74 (0.51-1.06)	102	0.63 (0.49-0.82)	0.84 (0.64-1.11)
Q4	227	30	0.27 (0.18-0.40)	0.47 (0.30-0.72)	92	0.54 (0.42-0.71)	0.69 (0.52-0.92)
P_{trend}			<0.0001	0.0005		<0.0001	0.0053
Intraepithelial PD-L1⁻ macrophage density							
Q1	228	57	1 (referent)	1 (referent)	111	1 (referent)	1 (referent)
Q2	228	57	1.00 (0.69-1.45)	1.22 (0.84-1.79)	107	0.97 (0.74-1.27)	1.14 (0.87-1.50)
Q3	227	52	0.95 (0.65-1.38)	0.98 (0.66-1.44)	110	1.02 (0.78-1.33)	1.09 (0.83-1.44)
Q4	227	76	1.43 (1.02-2.02)	1.31 (0.92-1.90)	121	1.18 (0.91-1.52)	1.11 (0.85-1.46)
P_{trend}			0.054	0.28		0.20	0.52
Stromal PD-L1⁻ macrophage density							
Q1	228	54	1 (referent)	1 (referent)	111	1 (referent)	1 (referent)
Q2	228	62	1.17 (0.81-1.68)	1.51 (1.03-2.20)	104	0.94 (0.72-1.23)	1.03 (0.79-1.35)
Q3	227	51	0.99 (0.68-1.46)	1.42 (0.95-2.11)	110	1.04 (0.80-1.36)	1.23 (0.94-1.61)
Q4	227	75	1.47 (1.04-2.09)	1.24 (0.87-1.77)	124	1.18 (0.92-1.53)	1.11 (0.86-1.44)

P_{trend}	0.065	0.34	0.14	0.26

The densities were divided into ordinal quartile categories from low (Q1) to high (Q4).
Multivariable Cox proportional hazards regression models were adjusted for sex (male, female), age (<65, 65–75, >75), year of operation (2000–2005, 2006–2010, 2011–2015), tumor location (proximal colon, distal colon, rectum), stage (I–II, III, IV), tumor grade (well/moderately differentiated, poorly differentiated), lymphovascular invasion (negative, positive), MMR status (proficient, deficient), and *BRAF* status (wild-type, mutant).
P_{trend} values were calculated by using the four categories of immune cell densities as continuous variables in univariable and multivariable Cox proportional hazard regression models.
Abbreviations: CI, confidence interval; HR, hazard ratio.

Table S5 Univariable and multivariable Cox regression models for cancer-specific and overall survival according to PD-L1 expression in tumor cells.

		Colorectal cancer-specific survival			Overall survival		
	No. of cases	No. of events	Univariable HR (95% CI)	Multivariable HR (95% CI)	No. of events	Univariable HR (95% CI)	Multivariable HR (95% CI)
PD-L1 negative	844	228	1 (referent)	1 (referent)	417	1 (referent)	1 (referent)
PD-L1 positive	66	14	0.78 (0.46-1.34)	0.93 (0.53-1.64)	32	0.96 (0.67-1.38)	0.83 (0.56-1.22)
P			0.37	0.80		0.83	0.34

Tumors were categorized into PD-L1 negative or positive using histoscore method with a cut-off >5 for the positivity.
Multivariable Cox proportional hazards regression models were adjusted for sex (male, female), age (<65, 65–75, >75), year of operation (2000–2005, 2006–2010, 2011–2015), tumor location (proximal colon, distal colon, rectum), stage (I–II, III, IV), tumor grade (well/moderately differentiated, poorly differentiated), lymphovascular invasion (negative, positive), MMR status (proficient, deficient), and *BRAF* status (wild-type, mutant).
Abbreviations: CI, confidence interval; HR, hazard ratio.

Table S6 Univariable and multivariable Cox regression models for cancer-specific and overall survival according to PD-L1⁺ and PD-L1⁻ macrophage densities in strata of MMR status.

		Colorectal cancer-specific survival			Overall survival		
	No. of cases	No. of events	Univariable HR (95% CI)	Multivariable HR (95% CI)	No. of events	Univariable HR (95% CI)	Multivariable HR (95% CI)
**MMR proficient**							
PD-L1⁺ macrophage density							
Low (Q1–Q2)	423	144	1 (referent)	1 (referent)	227	1 (referent)	1 (referent)
High (Q3–Q4)	350	74	0.57 (0.43-0.76)	0.74 (0.55-0.99)	153	0.74 (0.60-0.91)	0.81 (0.66-1.00)
P			0.0001	0.04		0.0043	0.05
**MMR deficient**							
PD-L1⁺ macrophage density							
Low (Q1–Q2)	33	10	1 (referent)	1 (referent)	18	1 (referent)	1 (referent)
High (Q3–Q4)	104	14	0.42 (0.19-0.95)	0.36 (0.14-0.93)	51	0.82 (0.48-1.40)	0.67 (0.38-1.19)
P			0.036	0.036		0.46	0.18
$P_{interaction}$			0.45	0.058		0.72	0.38
**MMR proficient**							
PD-L1⁻ macrophage density							
Low (Q1–Q2)	395	93	1 (referent)	1 (referent)	187	1 (referent)	1 (referent)
High (Q3–Q4)	378	125	1.46 (1.12-1.91)	1.12 (0.85-1.47)	193	1.13 (0.92-1.38)	0.95 (0.77-1.16)
P			0.0057	0.42		0.25	0.59
**MMR deficient**							
PD-L1⁻ macrophage density							
Low (Q1–Q2)	61	12	1 (referent)	1 (referent)	31	1 (referent)	1 (referent)
High (Q3–Q4)	76	12	0.80 (0.36-1.79)	0.79 (0.33-1.87)	38	1.03 (0.64-1.66)	1.00 (0.61-1.65)
P			0.59	0.59		0.89	1.00
$P_{interaction}$			0.18	0.24		0.71	0.94

The densities were divided into low (Q1–Q2) and high (Q3–Q4) using median as the cut-point.
Multivariable Cox proportional hazards regression models were adjusted for sex (male, female), age (<65, 65–75, >75), year of operation (2000–2005, 2006–2010, 2011–2015), tumor location (proximal colon, distal colon, rectum), stage (I–II, III, IV), tumor grade (well/moderately differentiated, poorly differentiated), lymphovascular invasion (negative, positive), and *BRAF* status (wild-type, mutant).
$P_{interaction}$ was calculated using the Wald test for the cross product of the immune cell density (low vs high) and MMR status (proficient vs deficient) in the Cox regression model.
Abbreviations: CI, confidence interval; HR, hazard ratio; MMR, mismatch repair

Table S7 Univariable and multivariable Cox regression models for cancer-specific and overall survival according to PD-L1⁺ and PD-L1⁻ macrophage densities in strata of AJCC stage.

		Colorectal cancer-specific survival			Overall survival		
	No. of cases	No. of events	Univariable HR (95% CI)	Multivariable HR (95% CI)	No. of events	Univariable HR (95% CI)	Multivariable HR (95% CI)
**AJCC stage (I)**							
PD-L1⁺ macrophage density							
Low (Q1–Q2)	63	7	1 (referent)	1 (referent)	22	1 (referent)	1 (referent)
High (Q3–Q4)	88	7	0.74 (0.26-2.11)	1.06 (0.34-3.32)	34	1.17 (0.68-2.00)	1.16 (0.65-2.08)
P			0.57	0.91		0.57	0.61
**AJCC stage (II)**							
PD-L1⁺ macrophage density							
Low (Q1–Q2)	141	24	1 (referent)	1 (referent)	61	1 (referent)	1 (referent)
High (Q3–Q4)	201	16	0.44 (0.23-0.83)	0.43 (0.22-0.84)	76	0.80 (0.57-1.12)	0.78 (0.54-1.11)
P			0.011	0.014		0.20	0.17
**AJCC stage (III)**							
PD-L1⁺ macrophage density							
Low (Q1–Q2)	175	59	1 (referent)	1 (referent)	91	1 (referent)	1 (referent)
High (Q3–Q4)	126	35	0.85 (0.56-1.29)	0.89 (0.56-1.39)	58	0.91 (0.66-1.27)	0.85 (0.59-1.21)
P			0.44	0.60		0.59	0.36
**AJCC stage (IV)**							
PD-L1⁺ macrophage density							
Low (Q1–Q2)	77	64	1 (referent)	1 (referent)	71	1 (referent)	1 (referent)
High (Q3–Q4)	39	30	0.87 (0.56-1.34)	0.67 (0.41-1.10)	36	0.93 (0.62-1.38)	0.68 (0.43-1.07)
P			0.53	0.11		0.71	0.09
**AJCC stage (I)**							
PD-L1⁻ macrophage density							
Low (Q1–Q2)	88	6	1 (referent)	1 (referent)	31	1 (referent)	1 (referent)
High (Q3–Q4)	63	8	1.86 (0.65-5.37)	2.20 (0.65-7.49)	25	0.12 (0.66-1.90)	0.89 (0.51-1.56)
P			0.25	0.21		0.67	0.69
**AJCC stage (II)**							
PD-L1⁻ macrophage density							
Low (Q1–Q2)	179	20	1 (referent)	1 (referent)	71	1 (referent)	1 (referent)
High (Q3–Q4)	163	20	1.11 (0.60-2.06)	1.08 (0.57-2.06)	66	1.03 (0.74-1.44)	1.07 (0.76-1.50)
P			0.75	0.81		0.86	0.71
**AJCC stage (III)**							
PD-L1⁻ macrophage density							
Low (Q1–Q2)	141	41	1 (referent)	1 (referent)	71	1 (referent)	1 (referent)
High (Q3–Q4)	160	53	1.16 (0.77-1.74)	1.14 (0.75-1.72)	78	0.99 (0.71-1.36)	0.96 (0.70-1.33)
P			0.48	0.54		0.93	0.82
**AJCC stage (IV)**							
PD-L1⁻ macrophage density							
Low (Q1–Q2)	48	38	1 (referent)	1 (referent)	45	1 (referent)	1 (referent)
High (Q3–Q4)	68	56	0.92 (0.61-1.38)	0.83 (0.53-1.30)	62	0.87 (0.59-1.28)	0.77 (0.50-1.17)
P			0.68	0.42		0.49	0.22

The densities were divided into low (Q1–Q2) and high (Q3–Q4) using median as the cut-point.
Multivariable Cox proportional hazards regression models were adjusted for sex (male, female), age (<65, 65–75, >75), year of operation (2000–2005, 2006–2010, 2011–2015), tumor location (proximal colon, distal colon, rectum), tumor grade (well/moderately differentiated, poorly differentiated), lymphovascular invasion (negative, positive), MMR status (proficient, deficient), and *BRAF* status (wild-type, mutant).
Abbreviations: AJCC, American Joint Committee on Cancer; CI, confidence interval; HR, hazard ratio.

Table S8 Univariable and multivariable Cox regression models for cancer-specific and overall survival according to PD-L1⁺ and PD-L1⁻ macrophage density in strata of AJCC stage.

		Colorectal cancer-specific survival			Overall survival		
	No. of cases	No. of events	Univariable HR (95% CI)	Multivariable HR (95% CI)	No. of events	Univariable HR (95% CI)	Multivariable HR (95% CI)
**Low AJCC stage (I–III)**							
PD-L1⁺ macrophage density							
Low (Q1–Q2)	379	90	1 (referent)	1 (referent)	174	1 (referent)	1 (referent)
High (Q3–Q4)	415	58	0.57 (0.41-0.79)	0.63 (0.45-0.90)	168	0.85 (0.68-1.05)	0.80 (0.64-1.00)
P			0.0008	0.010		0.12	0.058
**High AJCC stage (IV)**							
PD-L1⁺ macrophage density							
Low (Q1–Q2)	77	64	1 (referent)	1 (referent)	71	1 (referent)	1 (referent)
High (Q3–Q4)	39	30	0.87 (0.56-1.34)	0.67 (0.41-1.10)	36	0.93 (0.62-1.38)	0.68 (0.43-1.07)
P			0.53	0.11		0.71	0.094
$P_{interaction}$			0.13	0.57		0.73	0.65
**Low AJCC stage (I–III)**							
PD-L1⁻ macrophage density							
Low (Q1–Q2)	408	67	1 (referent)	1 (referent)	173	1 (referent)	1 (referent)
High (Q3–Q4)	386	81	1.31 (0.95-1.81)	1.26 (0.91-1.75)	169	1.06 (0.85-1.31)	1.00 (0.81-1.24)
P			0.11	0.16		0.62	0.97
**High AJCC stage (IV)**							
PD-L1⁻ macrophage density							
Low (Q1–Q2)	48	38	1 (referent)	1 (referent)	45	1 (referent)	1 (referent)
High (Q3–Q4)	68	56	0.92 (0.61-1.38)	0.83 (0.53-1.30)	62	0.87 (0.59-1.28)	0.77 (0.50-1.17)
P			0.68	0.42		0.49	0.22
$P_{interaction}$			0.19	0.16		0.45	0.62

The densities were divided into low (Q1–Q2) and high (Q3–Q4) using median as the cut-point.
Multivariable Cox proportional hazards regression models were adjusted for sex (male, female), age (<65, 65–75, >75), year of operation (2000–2005, 2006–2010, 2011–2015), tumor location (proximal colon, distal colon, rectum), tumor grade (well/moderately differentiated, poorly differentiated), lymphovascular invasion (negative, positive), MMR status (proficient, deficient), and *BRAF* status (wild-type, mutant).
$P_{interaction}$ was calculated using the Wald test for the cross product of the immune cell density (low vs high) and AJCC stage (I–III vs IV) in the Cox regression model.
Abbreviations: AJCC, American Joint Committee on Cancer; CI, confidence interval; HR, hazard ratio.

Table S9 Multivariable Cox regression models for cancer-specific survival according to PD-1+ and PD-1− T densities in the tumor center and the invasive margin with all variables.

	**PD-1+ T cell density**		**PD-1− T cell density**	
	Tumor center	Invasive margin	Tumor center	Invasive margin
	HR (95% CI)	HR (95% CI)	HR (95% CI)	HR (95% CI)
PD-1+ T cell density			Not included	Not included
Q1	1 (referent)	1 (referent)		
Q2	0.82 (0.58-1.16)	0.81 (0.58-1.14)		
Q3	0.75 (0.54-1.04)	0.83 (0.58-1.17)		
Q4	0.46 (0.30-0.71)	0.52 (0.34-0.79)		
PD-1− T cell density	Not included	Not included		
Q1			1 (referent)	1 (referent)
Q2			0.68 (0.48-0.96)	0.80 (0.58-1.12)
Q3			0.67 (0.48-0.94)	0.62 (0.43-0.90)
Q4			0.48 (0.32-0.70)	0.61 (0.42-0.88)
Age				
<65	1 (referent)	1 (referent)	1 (referent)	1 (referent)
65–75	1.13 (0.82-1.57)	1.14 (0.83-1.58)	1.16 (0.84-1.60)	1.18 (0.85-1.63)
>75	1.92 (1.38-2.67)	1.91 (1.38-2.65)	1.97 (1.42-2.73)	1.92 (1.39-2.66)
Sex				
Male	1 (referent)	1 (referent)	1 (referent)	1 (referent)
Female	0.88 (0.67-1.15)	0.86 (0.66-1.12)	0.88 (0.67-1.15)	0.90 (0.68-1.17)
Year of operation				
2000–2005	1 (referent)	1 (referent)	1 (referent)	1 (referent)
2006–2010	0.62 (0.45-0.85)	0.60 (0.44-0.82)	0.60 (0.44-0.82)	0.61 (0.45-0.83)
2011–2015	0.48 (0.34-0.66)	0.45 (0.32-0.66)	0.45 (0.32-0.62)	0.45 (0.32-0.62)
Tumor location				
Proximal colon	1 (referent)	1 (referent)	1 (referent)	1 (referent)
Distal colon	0.96 (0.72-1.29)	0.94 (0.70-1.26)	0.94 (0.70-1.26)	0.95 (0.71-1.26)
Rectum	0.81 (0.54-1.23)	0.81 (0.54-1.23)	0.82 (0.55-1.26)	0.80 (0.53-1.22)
AJCC stage				
I–II	1 (referent)	1 (referent)	1 (referent)	1 (referent)
III	2.68 (1.89-3.81)	2.70 (1.90-3.84)	2.81 (1.98-3.99)	2.79 (1.97-3.95)
IV	16.3 (11.2-23.7)	15.7 (10.8-22.9)	16.8 (11.6-24.5)	16.0 (11.0-23.2)
Tumor grade				
Low-grade (well to moderately differentiated)	1 (referent)	1 (referent)	1 (referent)	1 (referent)
High-grade (poorly differentiated)	1.95 (1.38-2.76)	1.99 (1.40-2.82)	1.96 (1.39-2.77)	1.88 (1.33-2.66)
Lymphovascular invasion				
No	1 (referent)	1 (referent)	1 (referent)	1 (referent)
Yes	1.95 (1.47-2.57)	2.01 (1.52-2.65)	2.01 (1.52-2.66)	2.03 (1.54-2.68)
MMR status				
MMR proficient	1 (referent)	1 (referent)	1 (referent)	1 (referent)
MMR deficient	0.59 (0.34-1.04)	1.58 (0.33-1.02)	0.56 (0.32-0.98)	0.54 (0.30-0.96)
BRAF mutation				
Wild-type	1 (referent)	1 (referent)	1 (referent)	1 (referent)
Mutant	1.29 (0.82-2.06)	1.31 (0.82-2.09)	1.23 (0.78-1.95)	1.34 (0.84-2.15)

The densities were divided into ordinal quartile categories from low (Q1) to high (Q4).
Abbreviations: AJCC, American Joint Committee on Cancer; CI, confidence interval; HR, hazard ratio; MMR, mismatch repair.

Table S10 Univariable and multivariable Cox regression models for cancer-specific and overall survival according to PD-1⁺ T cell densities in strata of PD-L1⁺ macrophage densities.

		Colorectal cancer-specific survival			Overall survival		
	No. of cases	No. of events	Univariable HR (95% CI)	Multivariable HR (95% CI)	No. of events	Univariable HR (95% CI)	Multivariable HR (95% CI)
**PD-L1⁺ macrophage density low (Q1–Q2)**							
PD-1⁺ T cell density							
Low (Q1–Q2)	337	122	1 (referent)	1 (referent)	197	1 (referent)	1 (referent)
High (Q3–Q4)	118	36	0.83 (0.58-1.21)	0.94 (0.63-1.40)	60	0.86 (0.64.1.15)	0.89 (0.66-1.21)
P			0.34	0.75		0.31	0.45
**PD-L1⁺ macrophage density high (Q3–Q4)**							
PD-1⁺ T cell density							
Low (Q1–Q2)	118	32	1 (referent)	1 (referent)	48	1 (referent)	1 (referent)
High (Q3–Q4)	337	52	0.55 (0.36-0.86)	0.72 (0.43-1.19)	144	0.99 (0.71-1.37)	0.99 (0.69-1.40)
P			0.0082	0.20		0.94	0.94
$P_{interaction}$			0.16	0.13		0.51	0.86

The densities of PD-1⁺ T cells and PD-L1⁺ macrophages were divided into low (Q1–Q2) and high (Q3–Q4) using median as the cut-point.
Multivariable Cox proportional hazards regression models were adjusted for sex (male, female), age (<65, 65–75, >75), year of operation (2000–2005, 2006–2010, 2011–2015), tumor location (proximal colon, distal colon, rectum), stage (I–II, III, IV), tumor grade (well/moderately differentiated, poorly differentiated), lymphovascular invasion (negative, positive), MMR status (proficient, deficient), and *BRAF* status (wild-type, mutant).
$P_{interaction}$ was calculated using the Wald test for the cross product of the macrophage density (low vs high) and T cell density (low vs high) in the Cox regression model.
Abbreviations: CI, confidence interval; HR, hazard ratio.

Table S11 Univariable and multivariable Cox regression models for cancer-specific and overall survival according PD-1$^+$ T cell densities in strata of PD-L1 expression in tumor cells.

		Colorectal cancer-specific survival			Overall survival		
	No. of cases	No. of events	Univariable HR (95% CI)	Multivariable HR (95% CI)	No. of events	Univariable HR (95% CI)	Multivariable HR (95% CI)
**Tumor cell PD-L1 positive**							
PD-1$^+$ T cell density							
Low (Q1–Q2)	5	2	1 (referent)	1 (referent)	2	1 (referent)	1 (referent)
High (Q3–Q4)	61	12	0.48 (0.11-2.14)	0.25 (0.02-3.28)	30	1.12 (0.27-4.68)	0.88 (0.17-4.52)
P			0.34	0.29		0.88	0.87
**Tumor cell PD-L1 negative**							
PD-1$^+$ T cell density							
Low (Q1–Q2)	450	152	1 (referent)	1 (referent)	243	1 (referent)	1 (referent)
High (Q3–Q4)	394	76	0.53 (0.40-0.69)	0.67 (0.50-0.90)	174	0.74 (0.61-0.90)	0.79 (0.64-0.97)
P			<0.0001	0.0071		0.0026	0.0023
$P_{interaction}$			0.83	0.55		0.57	0.75

The densities of PD-1$^+$ T cells were divided into low (Q1–Q2) and high (Q3–Q4) using median as the cut-point. Histoscore ≥5 was used as cut-off for PD-L1 positivity in tumors.

Multivariable Cox proportional hazards regression models were adjusted for sex (male, female), age (<65, 65–75, >75), year of operation (2000–2005, 2006–2010, 2011–2015), tumor location (proximal colon, distal colon, rectum), stage (I–II, III, IV), tumor grade (well/moderately differentiated, poorly differentiated), lymphovascular invasion (negative, positive), MMR status (proficient, deficient), and *BRAF* status (wild-type, mutant).

$P_{interaction}$ was calculated using the Wald test for the cross product of the PD-L1 expression in tumor cells (negative vs positive) and T cell density (low vs high) in the Cox regression model.

Abbreviations: CI, confidence interval; HR, hazard ratio.

Table S12 Univariable and multivariable Cox regression models for cancer-specific and overall survival according to the densities of M1-like and M2-like macrophages and their density ratio in the tumor center and the invasive margin.

		Colorectal cancer-specific survival			Overall survival		
	No. of cases	No. of events	Univariable HR (95% CI)	Multivariable HR (95% CI)	No. of events	Univariable HR (95% CI)	Multivariable HR (95% CI)
**Tumor center**							
M1-like macrophage density							
Q1	228	85	1 (referent)	1 (referent)	137	1 (referent)	1 (referent)
Q2	228	55	0.54 (0.38-0.75)	0.59 (0.42-0.83)	102	0.61 (0.47-0.79)	0.69 (0.54-0.90)
Q3	227	53	0.50 (0.36-0.71)	0.55 (0.38-0.77)	105	0.62 (0.48-0.81)	0.62 (0.45-0.80)
Q4	227	49	0.50 (0.35-0.71)	0.54 (0.38-0.78)	105	0.64 (0.50-0.83)	0.66 (0.51-0.86)
P_{trend}			<0.0001	0.0004		0.0013	0.0007
M2-like macrophage density							
Q1	228	48	1 (referent)	1 (referent)	99	1 (referent)	1 (referent)
Q2	228	58	1.27 (0.86-1.86)	0.83 (0.56-1.23)	112	1.28 (0.98-1.68)	0.97 (0.74-1.28)
Q3	227	64	1.44 (0.99-2.09)	1.03 (0.70-1.51)	107	1.21 (0.92-1.60)	1.03 (0.78-1.36)
Q4	227	72	1.69 (1.18-2.44)	1.10 (0.75-1.60)	131	1.59 (1.22-2.06)	1.13 (0.78-1.36)
P_{trend}			0.003	0.31		0.0014	0.29
M1:M2-like macrophage density ratio							
Q1	228	83	1 (referent)	1 (referent)	141	1 (referent)	1 (referent)
Q2	228	55	0.60 (0.43-0.84)	0.61 (0.43-0.86)	98	0.60 (0.46-0.78)	0.60 (0.46-0.78)
Q3	227	64	0.71 (0.51-0.98)	0.86 (0.62-1.20)	114	0.73 (0.57-0.94)	0.87 (0.68-1.12)
Q4	227	40	0.40 (0.27-0.58)	0.59 (0.40-0.87)	96	0.54 (0.41-0.69)	0.69 (0.53-0.91)
P_{trend}			<0.0001	0.040		<0.0001	0.069
**Invasive margin**							
M1-like macrophage density							
Q1	228	87	1 (referent)	1 (referent)	143	1 (referent)	1 (referent)
Q2	228	48	0.48 (0.34-0.69)	0.63 (0.44-0.91)	109	0.65 (0.51-0.84)	0.73 (0.57-0.94)
Q3	227	60	0.61 (0.44-0.84)	0.78 (0.56-1.10)	102	0.61 (0.47-0.79)	0.68 (0.53-0.88)
Q4	227	47	0.46 (0.32-0.65)	0.60 (0.41-0.86)	95	0.54 (0.42-0.70)	0.63 (0.49-0.83)
P_{trend}			<0.0001	0.014		<0.0001	0.0006
M2-like macrophage density							
Q1	228	57	1 (referent)	1 (referent)	101	1 (referent)	1 (referent)
Q2	228	55	0.98 (0.67-1.42)	1.07 (0.73-1.57)	109	1.13 (0.86-1.47)	1.17 (0.89-1.54)
Q3	227	52	0.99 (0.68-1.44)	1.03 (0.70-1.50)	108	1.14 (0.87-1.49)	1.21 (0.92-1.59)
Q4	227	78	1.49 (1.06-2.10)	1.14 (0.80-1.63)	131	1.47 (1.13-1.90)	1.22 (0.93-1.60)
P_{trend}			0.024	0.53		0.0051	0.15
M1:M2-like macrophage density ratio							
Q1	228	78	1 (referent)	1 (referent)	137	1 (referent)	1 (referent)
Q2	228	65	0.80 (0.58-1.11)	0.78 (0.56-1.10)	113	0.79 (0.62-1.01)	0.79 (0.61-1.01)
Q3	227	47	0.55 (0.38-0.78)	0.75 (0.52-1.09)	102	0.66 (0.51-0.86)	0.79 (0.61-1.03)
Q4	227	52	0.59 (0.42-0.84)	0.73 (0.51-1.05)	96	0.60 (0.47-0.79)	0.68 (0.52-0.89)
P_{trend}			0.0005	0.081		<0.0001	0.0074

The densities were divided into ordinal quartile categories from low (Q1) to high (Q4).
Multivariable Cox proportional hazards regression models were adjusted for sex (male, female), age (<65, 65–75, >75), year of operation (2000–2005, 2006–2010, 2011–2015), tumor location (proximal colon, distal colon, rectum), stage (I–II, III, IV), tumor grade (well/moderately differentiated, poorly differentiated), lymphovascular invasion (negative, positive), MMR status (proficient, deficient), and *BRAF* status (wild-type, mutant).
P_{trend} values were calculated by using the four categories of immune cell densities as continuous variables in univariable and multivariable Cox proportional hazard regression models.
Abbreviations: CI, confidence interval; HR, hazard ratio.

Table S13 Univariable and multivariable Cox regression models for cancer-specific survival for the densities of macrophage subtypes categorized according to the PD-L1 expression and M1/M2 polarization state in the tumor center and the invasive margin.

		**PD-L1⁺ macrophage density** Colorectal cancer-specific survival			**PD-L1⁻ macrophage density** Colorectal cancer-specific survival		
	No. of cases	No. of events	Univariable HR (95% CI)	Multivariable HR (95% CI)	No. of events	Univariable HR (95% CI)	Multivariable HR (95% CI)
**Tumor center**							
M1-like macrophages							
Q1	228	86	1 (referent)	1 (referent)	81	1 (referent)	1 (referent)
Q2	228	70	0.73 (0.53-1.00)	0.94 (0.68-1.30)	48	0.52 (0.36-0.74)	0.54 (0.38-0.78)
Q3	227	446	0.45 (0.32-0.65)	0.58 (0.40-0.83)	62	0.67 (0.48-0.93)	0.67 (0.48-0.94)
Q4	227	40	0.40 (0.27-0.58)	0.57 (0.39-0.85)	51	0.55 (0.38-0.78)	0.60 (0.42-0.86)
P_{trend}			<0.0001	0.0005		0.0034	0.011
M2-like macrophages							
Q1	340	104	1 (referent)	1 (referent)	48	1 (referent)	1 (referent)
Q2	190	49	0.85 (0.60-1.19)	0.99 (0.70-1.40)	50	1.10 (0.74-1.63)	0.75 (0.50-1.13)
Q3	190	47	0.77 (0.54-1.08)	0.85 (0.59-1.20)	67	1.59 (1.10-2.30)	1.01 (0.69-1.49)
Q4	190	42	0.69 (0.48-0.98)	0.81 (0.56-1.18)	77	1.84 (1.28-2.64)	1.17 (0.81-1.71)
P_{trend}			0.025	0.21		0.0001	0.11
M1/M2 mixed phenotype macrophages							
Q1	228	78	1 (referent)	1 (referent)	59	1 (referent)	1 (referent)
Q2	228	66	0.82 (0.59-1.13)	0.74 (0.52-1.03)	51	0.83 (0.57-1.21)	0.75 (0.51-1.10)
Q3	227	57	0.68 (0.49-0.96)	0.95 (0.67-1.35)	60	1.03 (0.72-1.47)	0.75 (0.52-1.09)
Q4	227	41	0.47 (0.32-0.68)	0.55 (0.37-0.82)	72	1.27 (0.90-1.80)	0.93 (0.65-1.32)
P_{trend}			<0.0001	0.017		0.089	0.79
**Invasive margin**							
M1-like macrophages							
Q1	228	93	1 (referent)	1 (referent)	76	1 (referent)	1 (referent)
Q2	228	69	0.72 (0.53-0.99)	1.07 (0.77-1.49)	50	0.59 (0.41-0.84)	0.57 (0.39-0.83)
Q3	227	50	0.48 (0.34-0.68)	0.69 (0.48-0.99)	53	0.60 (0.43-0.86)	0.82 (0.57-1.16)
Q4	227	30	0.27 (0.18-0.41)	0.51 (0.33-0.79)	63	0.76 (0.54-1.06)	0.72 (0.51-1.02)
P_{trend}			<0.0001	0.0008		0.12	0.20
M2-like macrophages							
Q1	259	82	1 (referent)	1 (referent)	55	1 (referent)	1 (referent)
Q2	217	65	0.91 (0.66-1.26)	1.17 (0.84-1.64)	52	0.99 (0.68-1.44)	1.11 (0.75-1.64)
Q3	217	55	0.82 (0.58-1.15)	0.97 (0.69-1.38)	54	1.05 (0.72-1.53)	1.09 (0.74-1.60)
Q4	217	40	0.54 (0.37-0.78)	0.68 (0.46-1.01)	81	1.65 (1.17-2.32)	1.18 (0.83-1.68)
P_{trend}			0.0013	0.068		0.0036	0.40
M1/M2 mixed phenotype macrophages							
Q1	228	89	1 (referent)	1 (referent)	58	1 (referent)	1 (referent)
Q2	228	70	0.77 (0.56-1.05)	1.13 (0.82-1.56)	56	0.96 (0.67-1.39)	1.07 (0.73-1.56)
Q3	227	46	0.47 (0.33-0.68)	0.74 (0.51-1.07)	54	0.93 (0.64-1.35)	1.09 (0.75-1.60)
Q4	227	37	0.37 (0.25-0.54)	0.57 (0.38-0.86)	74	1.29 (0.92-1.82)	1.07 (0.76-1.52)
P_{trend}			<0.0001	0.0029		0.16	0.70

The densities were divided into ordinal categories from low (Q1) to high (Q4).
Multivariable Cox proportional hazards regression models were adjusted for sex (male, female), age (<65, 65–75, >75), year of operation (2000–2005, 2006–2010, 2011–2015), tumor location (proximal colon, distal colon, rectum), stage (I–II, III, IV), tumor grade (well/moderately differentiated, poorly differentiated), lymphovascular invasion (negative, positive), MMR status (proficient, deficient), and *BRAF* status (wild-type, mutant).
P_{trend} values were calculated by using the four categories of immune cell densities as continuous variables in univariable and multivariable Cox proportional hazard regression models.
Abbreviations: CI, confidence interval; HR, hazard ratio.

Supplementary figures

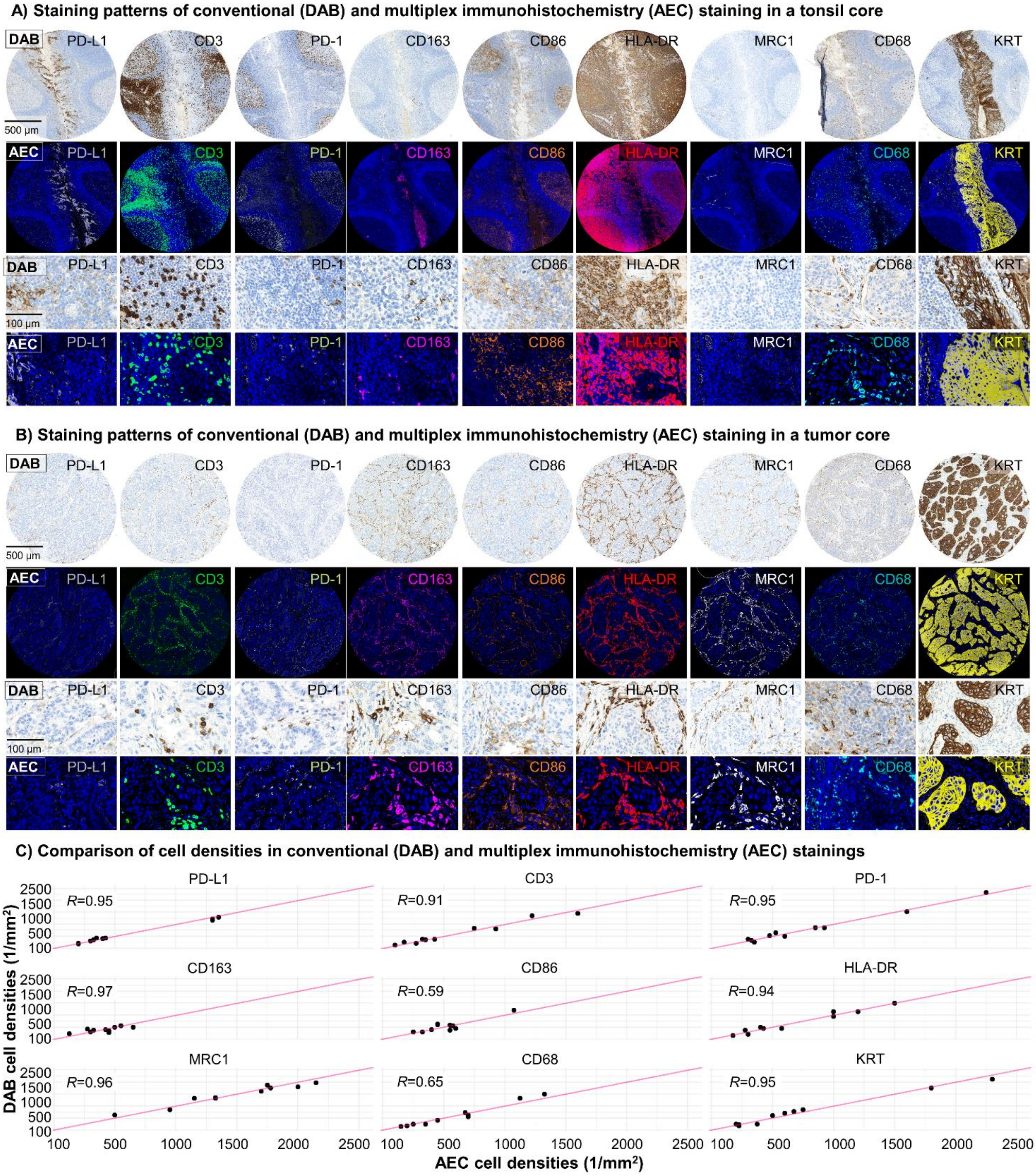

Figure S1 Comparison of staining patterns in conventional and multiplex immunohistochemistry. Examples of the staining patterns in 10 serial sections (9 sections for conventional immunohistochemistry and 1 for multiplex immunohistochemistry) of one tonsil core (A) and 10 serial sections of one tumor core (B). Correlations between cell densities in conventional and multiplex immunohistochemistry stainings calculated from 10 tumor regions (size 200 x 200 µm) for each marker. The correlations were measured using Spearman's rank correlation coefficients (*R*).

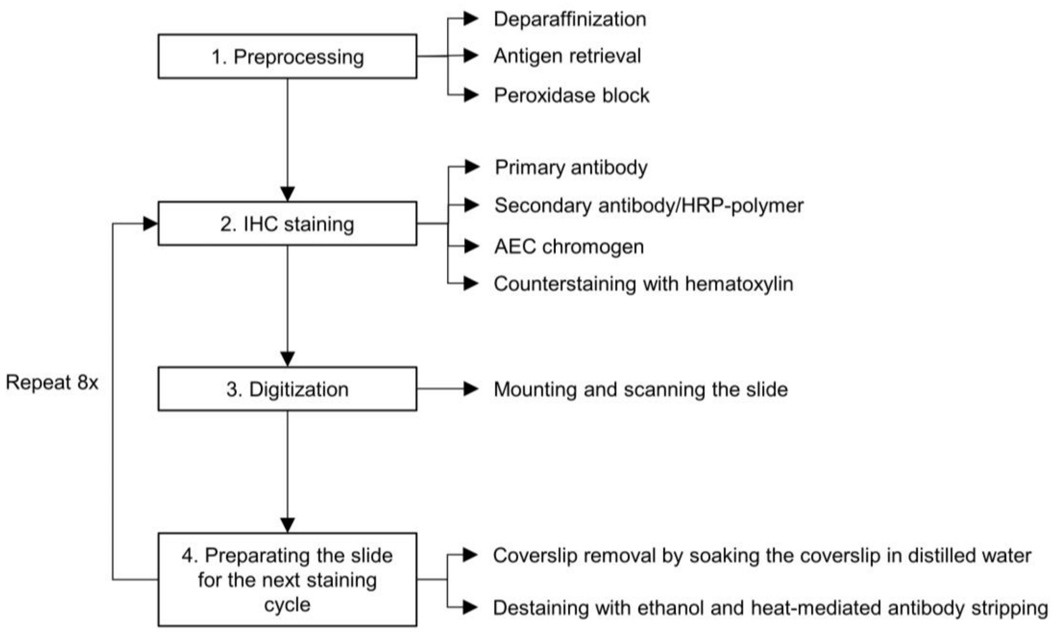

Figure S2 Multiplex immunohistochemistry staining procedure. Each slide was stained 9 times sequentially using an automated IHC stainer.

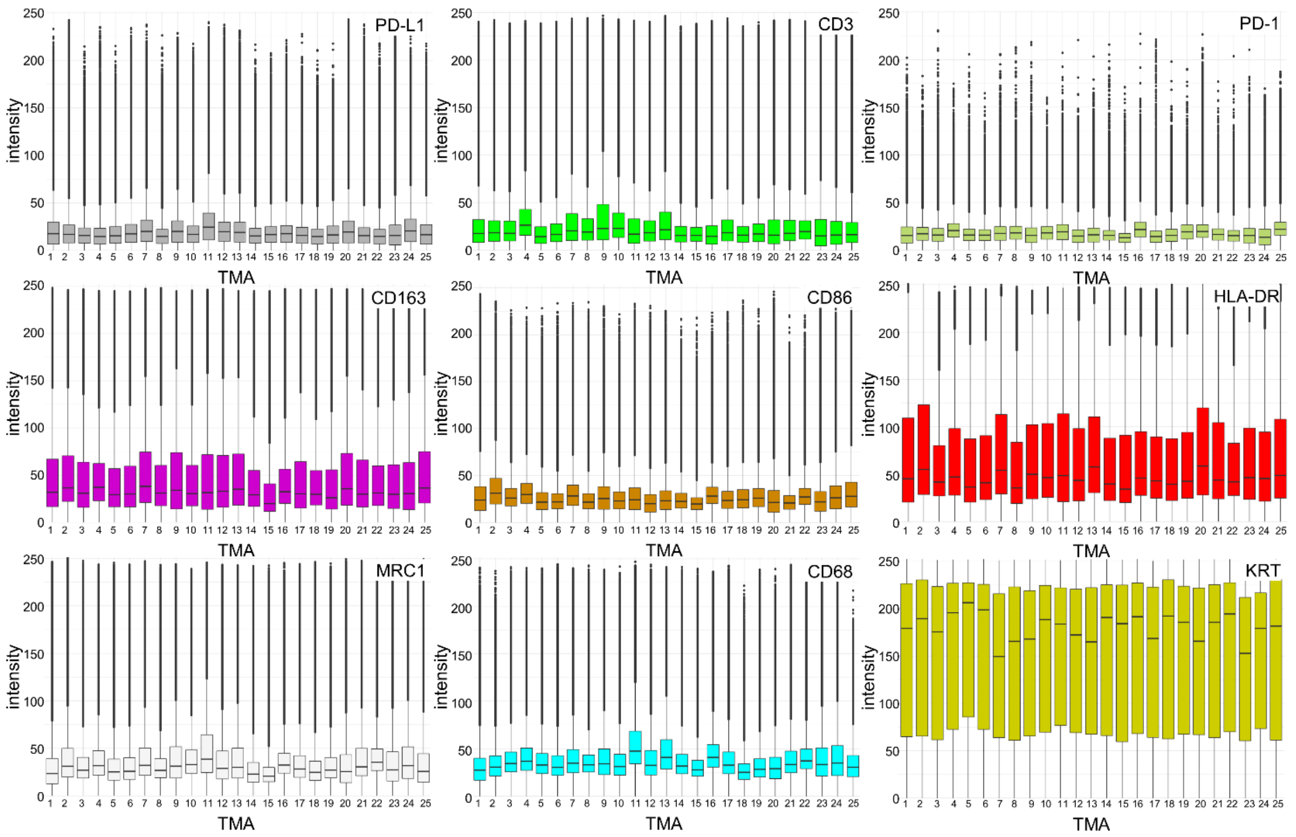

Figure S3 Staining signal intensities for multiplex immunohistochemistry markers across tissue microarrays. The boxplots represent intensities across all cells in 25 tissue microarrays included in the study.

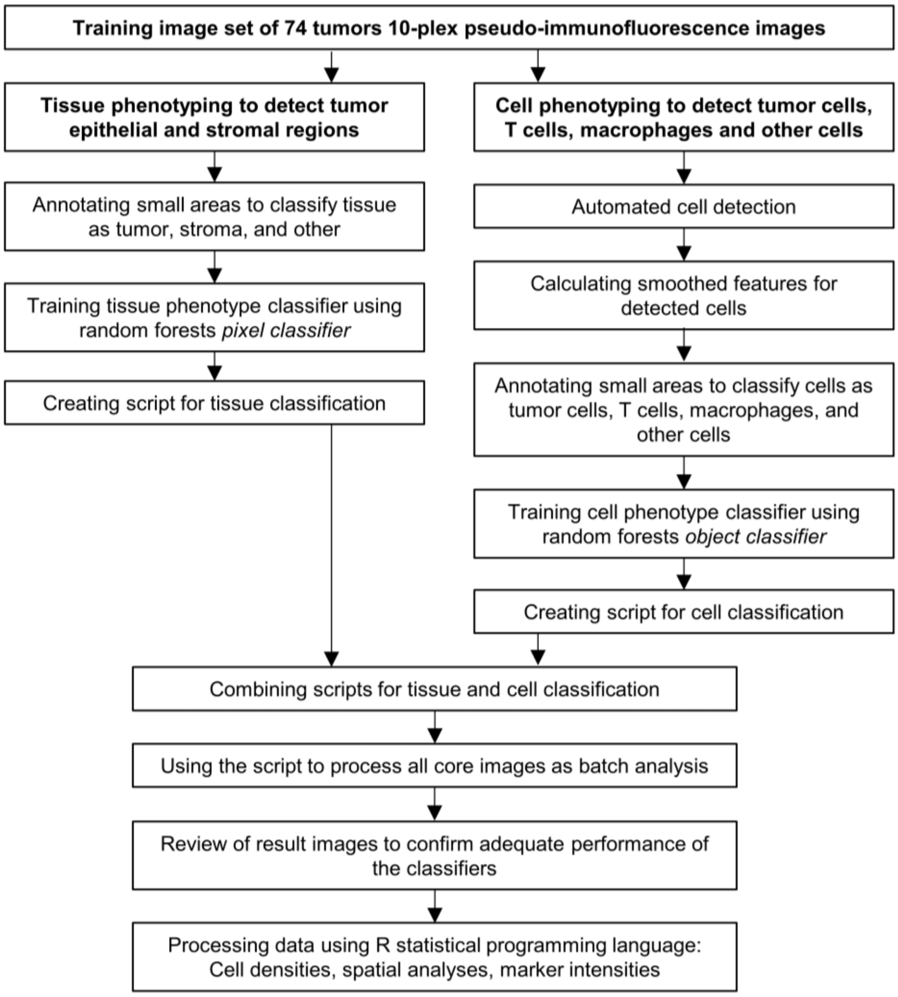

Figure S4 Flowchart for cell and tissue analysis of the multiplex-immunohistochemistry images in QuPath-software.

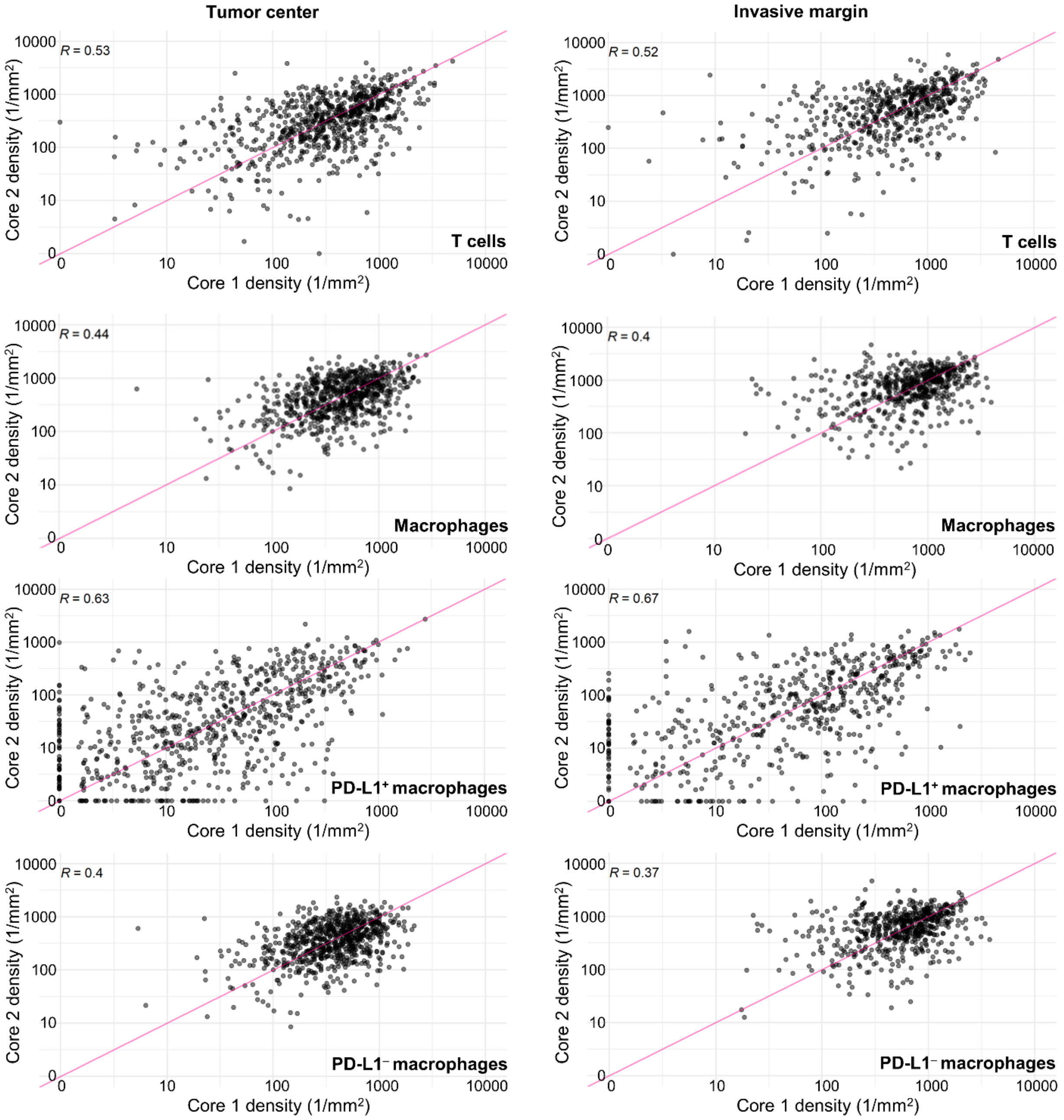

Figure S5 Core to core-correlations for T cell, macrophage and PD-L1⁺ and PD-L1⁻ macrophage densities in the tumor center and the invasive margin. Correlations are calculated in two randomly chosen cores of tumors with two or more cores measured using Spearman's rank correlation coefficients (*R*). The correlations are represented separately for the tumor center and the invasive margin. N=761 in the tumor center and N=589 in the invasive margin.

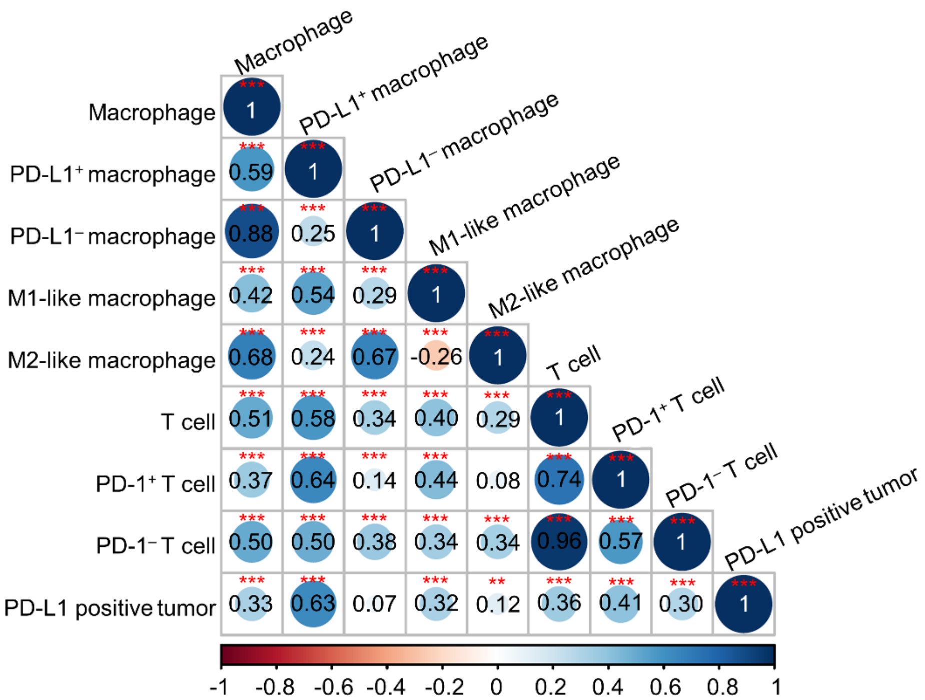

Figure S6 Spearman correlation coefficients between the total immune cell densities. Statistically significant correlations are shown with asterisks (****P*<0.0001, ***P*<0.001,* P<0.005).

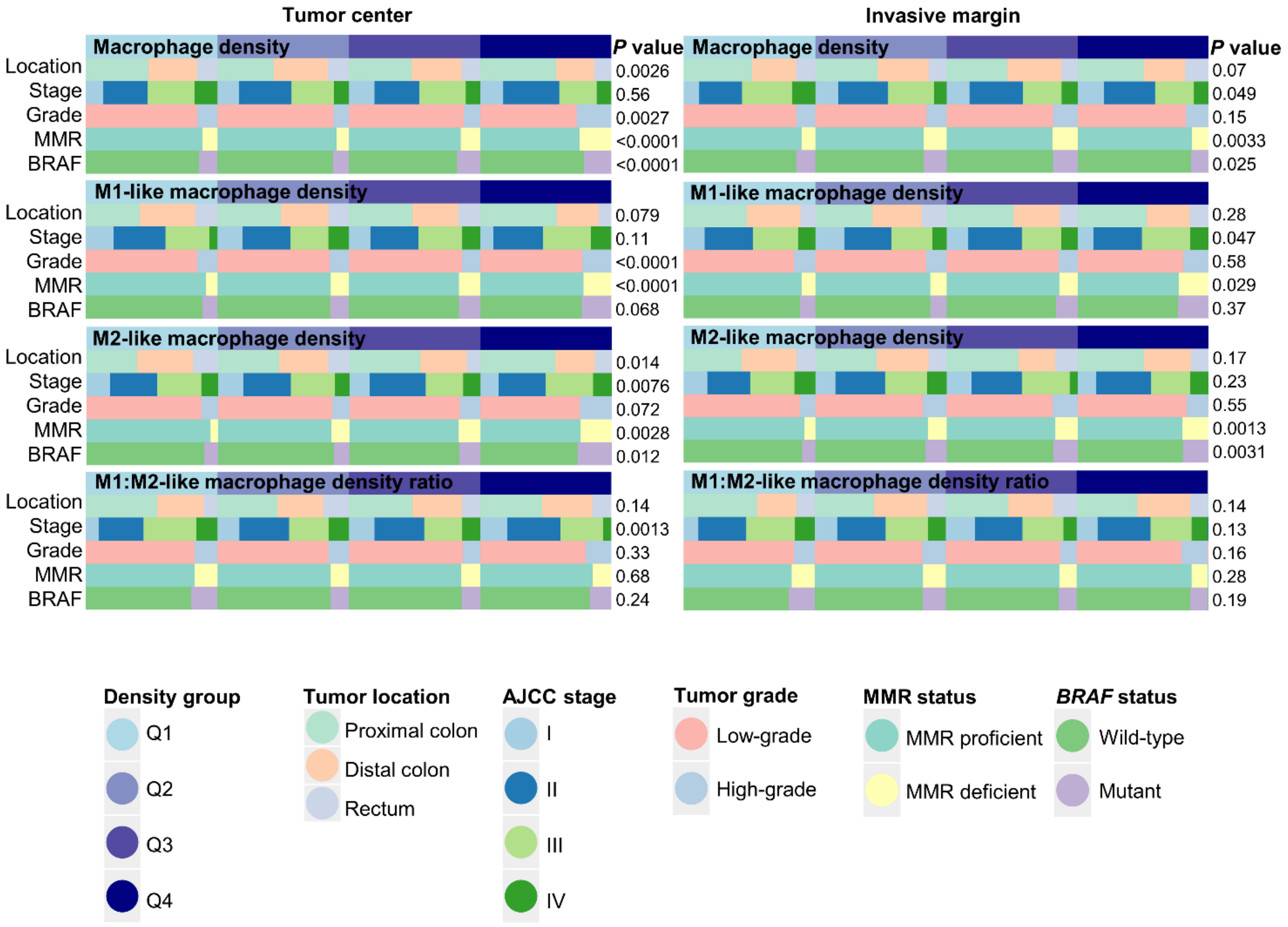

Figure S7 The clinicopathologic features in relation to total, M1-like, and M2-like macrophage densities and to the density ratio of M1-like and M2-like macrophages in the tumor center and the invasive margin. The densities were divided into ordinal quartiles from low (Q1) to high (Q4). The statistical significance was determined with Chi-square test. Abbreviations: AJCC, American Joint Committee on Cancer; MMR, mismatch repair.

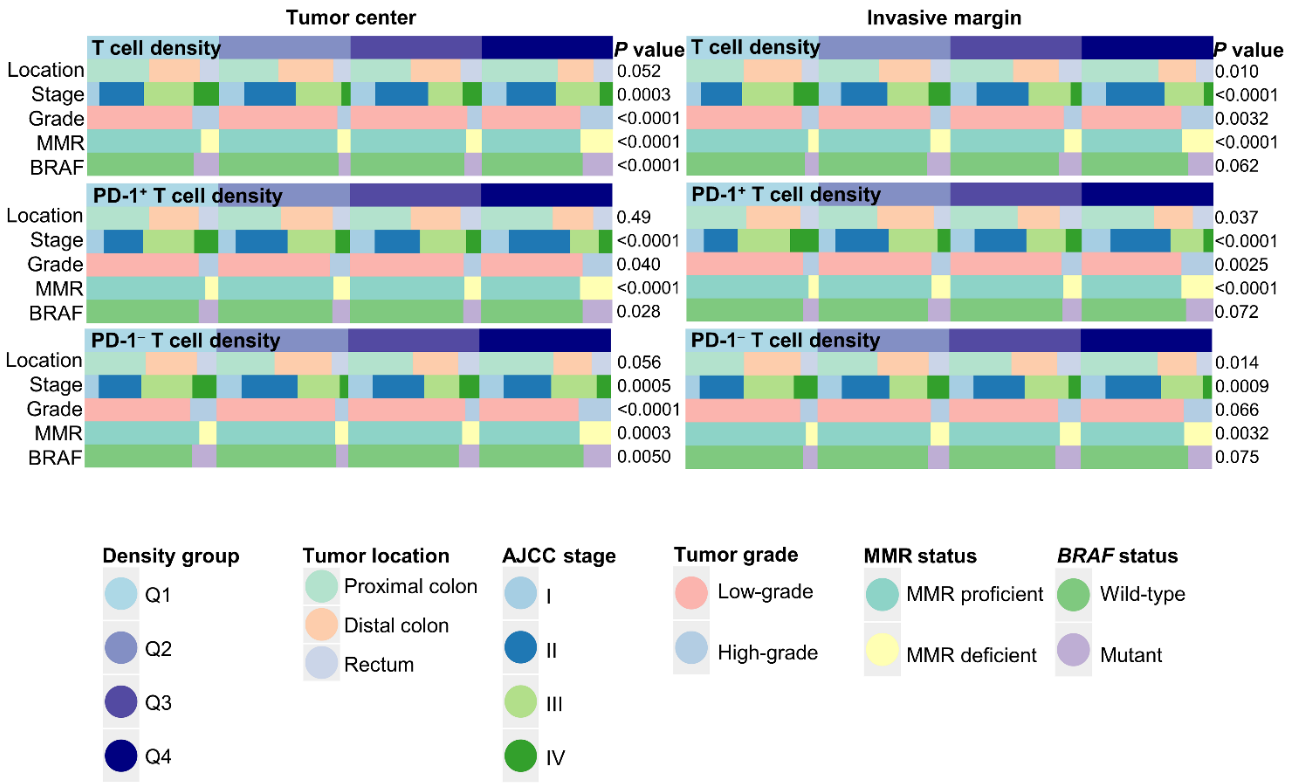

Figure S8 The clinicopathologic features in relation to total, PD-1$^+$ and PD-1$^-$ T cell densities in the tumor center and the invasive margin. The densities were divided into ordinal quartiles from low (Q1) to high (Q4). The statistical significance was determined with Chi-square test. Abbreviations: AJCC, American Joint Committee on Cancer; MMR, mismatch repair.

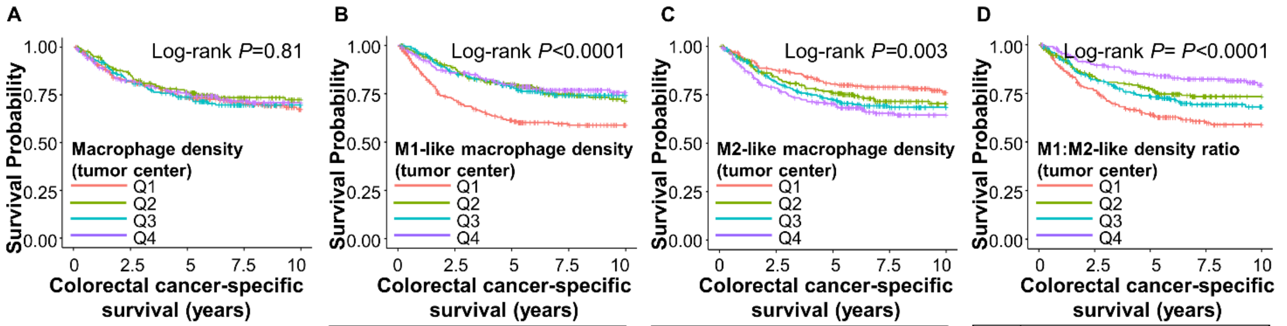

	Years, number at risk				
A	0	2.5	5	7.5	10
Q1	228	169	240	103	61
Q2	228	181	145	92	59
Q3	227	173	138	86	63
Q4	227	170	141	92	59

	Years, number at risk				
B	0	2.5	5	7.5	10
Q1	228	145	111	78	54
Q2	228	187	153	99	71
Q3	227	181	148	98	65
Q4	227	180	152	98	52

	Years, number at risk				
C	0	2.5	5	7.5	10
Q1	228	190	159	121	61
Q2	228	173	139	81	54
Q3	227	169	137	91	72
Q4	227	161	129	80	55

	Years, number at risk				
D	0	2.5	5	7.5	10
Q1	228	159	117	70	50
Q2	228	171	147	100	81
Q3	227	172	136	81	52
Q4	227	191	164	122	59

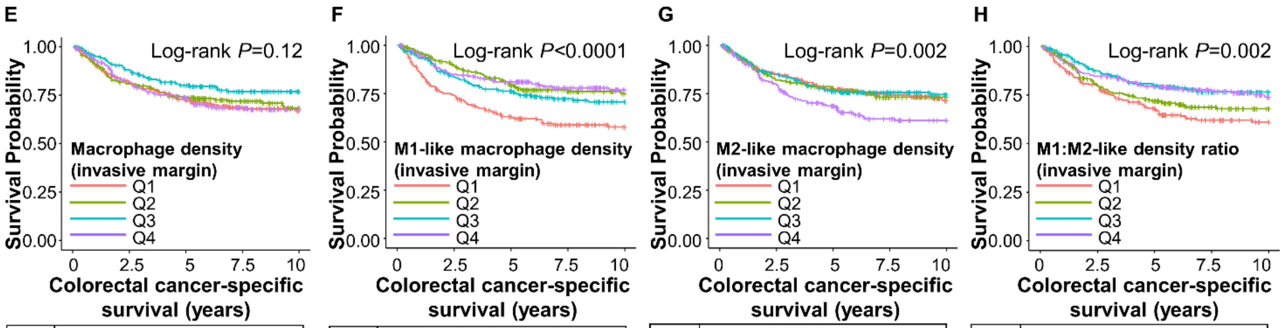

	Years, number at risk				
E	0	2.5	5	7.5	10
Q1	228	169	138	90	60
Q2	228	168	141	88	49
Q3	227	186	149	100	67
Q4	227	170	136	95	66

	Years, number at risk				
F	0	2.5	5	7.5	10
Q1	228	156	114	75	45
Q2	228	180	142	91	65
Q3	227	175	149	97	71
Q4	227	182	159	110	61

	Years, number at risk				
G	0	2.5	5	7.5	10
Q1	228	181	152	107	58
Q2	228	170	146	91	62
Q3	227	176	142	90	58
Q4	227	166	124	85	64

	Years, number at risk				
H	0	2.5	5	7.5	10
Q1	228	166	123	84	58
Q2	228	163	135	85	59
Q3	227	177	149	95	67
Q4	227	185	156	108	57

Figure S9 Kaplan-Meier cancer-specific survival curves for the densities of total, M1-like, and M2-like macrophages and for the density ratio of M1-like and M2-like macrophages in the tumor center and the invasive margin. The densities were divided into ordinal quartiles from low (Q1) to high (Q4). Statistical significance was determined with Log-rank test.

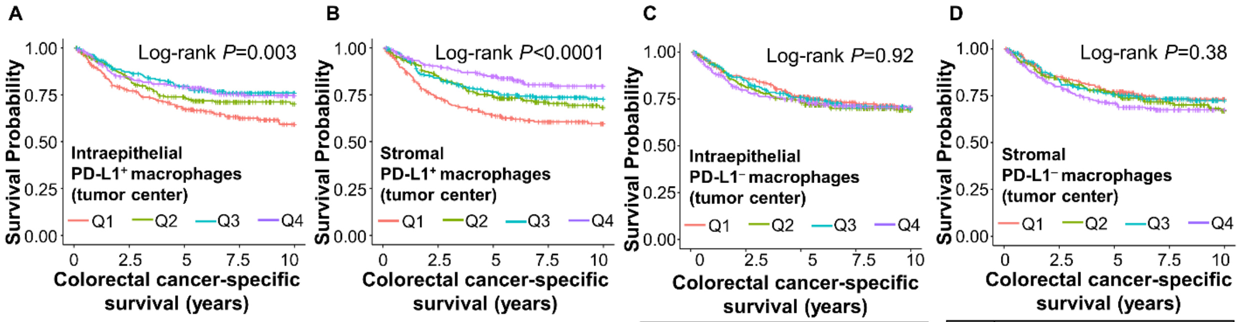

A

	Years, number at risk				
	0	2.5	5	7.5	10
Q1	228	161	128	82	45
Q2	228	178	141	95	72
Q3	227	181	145	100	63
Q4	227	173	150	96	62

B

	Years, number at risk				
	0	2.5	5	7.5	10
Q1	228	150	120	82	52
Q2	228	180	140	89	59
Q3	227	176	145	105	65
Q4	227	187	159	97	66

C

	Years, number at risk				
	0	2.5	5	7.5	10
Q1	228	185	145	96	60
Q2	228	172	145	107	64
Q3	227	173	134	79	56
Q4	227	163	140	91	62

D

	Years, number at risk				
	0	2.5	5	7.5	10
Q1	228	176	147	98	63
Q2	228	177	142	91	57
Q3	227	172	142	102	65
Q4	227	168	133	82	57

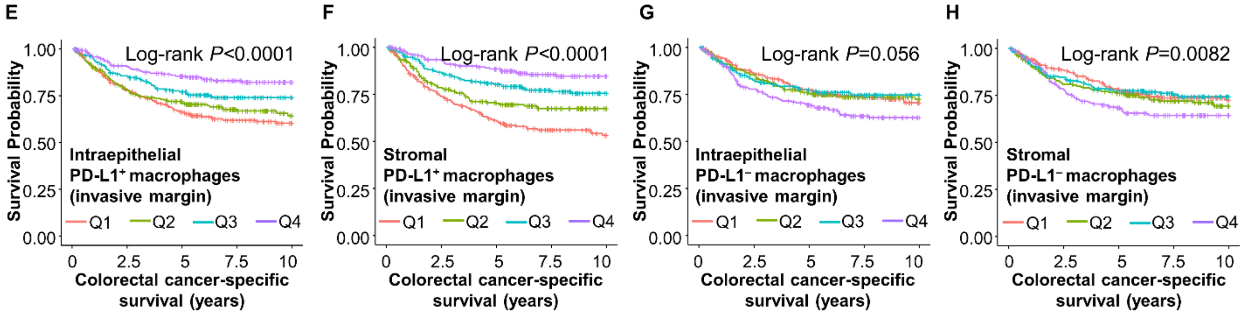

E

	Years, number at risk				
	0	2.5	5	7.5	10
Q1	233	169	133	93	61
Q2	226	163	132	81	48
Q3	225	179	142	89	60
Q4	226	182	157	110	72

F

	Years, number at risk				
	0	2.5	5	7.5	10
Q1	228	155	118	78	51
Q2	228	164	128	88	57
Q3	227	183	152	98	62
Q4	227	191	166	109	72

G

	Years, number at risk				
	0	2.5	5	7.5	10
Q1	228	179	143	98	60
Q2	228	185	146	95	57
Q3	227	168	143	96	64
Q4	227	161	132	84	61

H

	Years, number at risk				
	0	2.5	5	7.5	10
Q1	228	182	146	92	61
Q2	228	173	154	100	61
Q3	227	172	138	87	56
Q4	227	166	126	94	64

Figure S10 Kaplan-Meier cancer-specific survival curves for the PD-L1⁺ and PD-L1⁻ macrophage densities in tumor intraepithelial and stromal regions of the tumor center and the invasive margin. The densities were divided into ordinal quartiles from low (Q1) to high (Q4). Statistical significance was determined with Log-rank test.

A

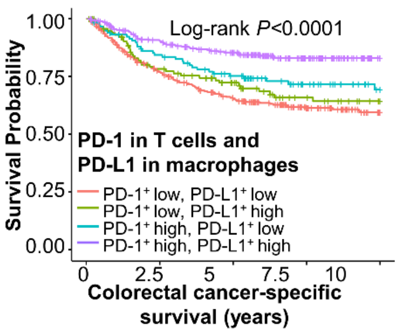

	Years, number at risk				
	0	2.5	5	7.5	10
PD-1+ low, PD-L1+ low	338	235	183	124	81
PD-1+ low, PD-L1+ high	118	83	64	42	30
PD-1+ high, PD-L1+ low	118	95	78	49	29
PD-1+ high, PD-L1+ high	336	280	239	158	102

B

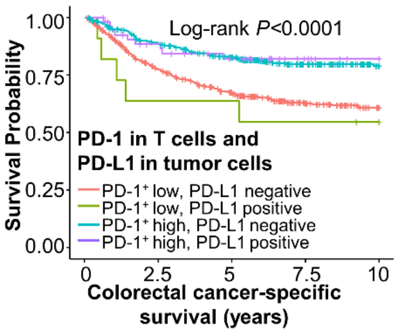

	Years, number at risk				
	0	2.5	5	7.5	10
PD-1+ low, PD-L1 negative	445	311	240	160	106
PD-1+ low, PD-L1 positive	11	7	7	6	5
PD-1+ high, PD-L1 negative	399	332	281	184	114
PD-1+ high, PD-L1 positive	55	43	36	23	17

Figure S11 Kaplan-Meier cancer-specific survival curves according to expression levels of PD-L1 and PD-1. A, tumors divided into four groups according to low (Q1–Q2) and high (Q3–Q4) density of PD-1$^+$ T cells and low (Q1–Q2) and high (Q3–Q4) density of PD-L1$^+$ macrophages. **B**, tumors divided into four groups according to low (Q1–Q2) and high (Q3–Q4) density of PD-1$^+$ T cells and PD-L1 negativity and positivity in tumor cells. Statistical significance was determined with Log-rank test.

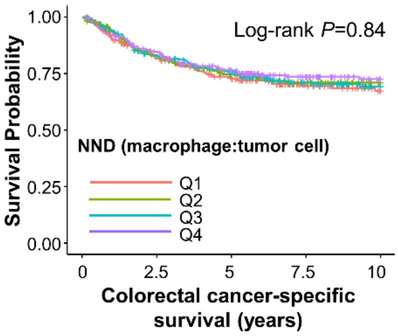

	Years, number at risk				
	0	2.5	5	7.5	10
Q1	228	166	139	85	59
Q2	228	175	139	97	64
Q3	227	173	147	97	59
Q4	227	179	139	94	60

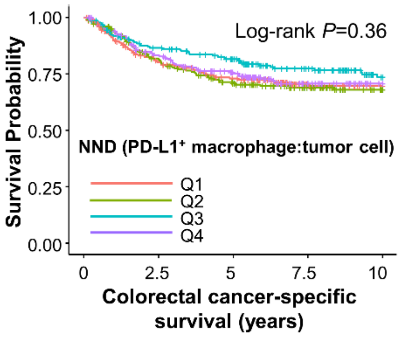

	Years, number at risk				
	0	2.5	5	7.5	10
Q1	224	162	135	85	61
Q2	224	167	131	89	56
Q3	223	180	153	108	64
Q4	223	171	137	85	57

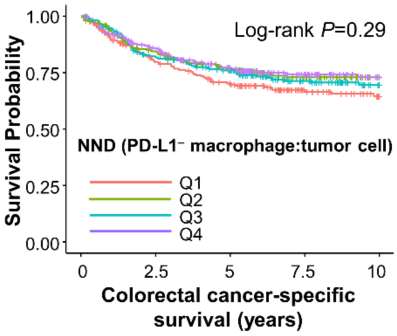

	Years, number at risk				
	0	2.5	5	7.5	10
Q1	228	162	133	80	55
Q2	228	179	145	102	69
Q3	227	173	148	98	60
Q4	227	179	138	93	58

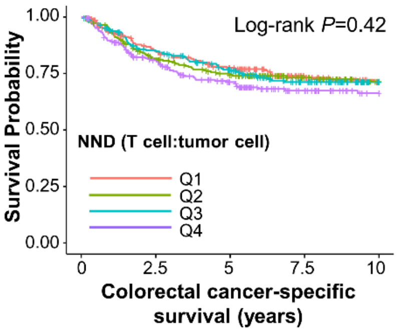

	Years, number at risk				
	0	2.5	5	7.5	10
Q1	228	175	149	96	67
Q2	228	176	149	98	65
Q3	227	177	141	98	54
Q4	227	165	125	81	56

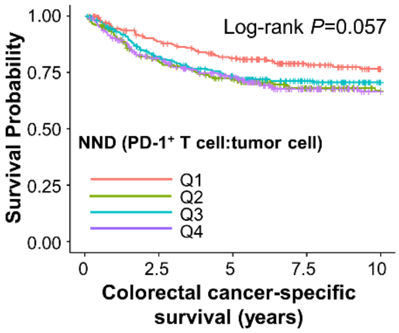

	Years, number at risk				
	0	2.5	5	7.5	10
Q1	227	185	156	108	73
Q2	227	167	140	82	57
Q3	226	171	138	103	61
Q4	226	166	127	78	49

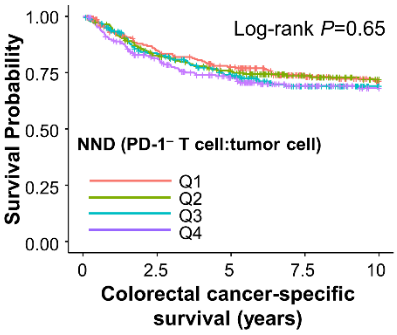

	Years, number at risk				
	0	2.5	5	7.5	10
Q1	228	175	149	96	65
Q2	228	178	151	98	67
Q3	227	172	135	95	52
Q4	227	168	129	84	58

Figure S12 Kaplan-Meier cancer-specific survival curves for the nearest neighbor distance analyses of immune cells. Nearest neigbor distance was calculated from each immune cell into the closest tumor cell. The average distances of each tumor were divided into ordinal quartiles from short (Q1) to long (Q4). The significance was tested with Log-rank test.
